# Buffer choice and pH strongly influence phase separation of SARS-CoV-2 nucleocapsid with RNA

**DOI:** 10.1091/mbc.E23-12-0500

**Published:** 2024-04-19

**Authors:** Nina C. Kathe, Mihajlo Novakovic, Frédéric H.-T. Allain

**Affiliations:** a Department of Biology, Institute of Biochemistry, ETH Zurich, Zurich, Switzerland; University of Texas Southwestern Medical Center

## Abstract

The SARS-CoV-2 nucleocapsid (N) protein is crucial for virus replication and genome packaging. N protein forms biomolecular condensates both in vitro and in vivo in a process known as liquid–liquid phase separation (LLPS), but the exact factors regulating LLPS of N protein are not fully understood. Here, we show that pH and buffer choice have a profound impact on LLPS of N protein. The degree of phase separation is highly dependent on the pH of the solution, which is correlated with histidine protonation in N protein. Specifically, we demonstrate that protonation of H356 is essential for LLPS in phosphate buffer. Moreover, electrostatic interactions of buffer molecules with specific amino acid residues are able to alter the net charge of N protein, thus influencing its ability to undergo phase separation in the presence of RNA. Overall, these findings reveal that even subtle changes in amino acid protonation or surface charge caused by the pH and buffer system can strongly influence the LLPS behavior, and point to electrostatic interactions as the main driving forces of N protein phase separation. Further, our findings emphasize the importance of these experimental parameters when studying phase separation of biomolecules, especially in the context of viral infections where the intracellular milieu undergoes drastic changes and intracellular pH normally decreases.

## Abstract (executive summary)

SARS-CoV-2 N protein undergoes liquid–liquid phase separation (LLPS) with RNA which is hypothe­sized to be important for viral genome packaging.

External factors such as buffer molecules and pH can profoundly influence the LLPS by interacting with charged residues and altering protein net charge. For example, enhanced LLPS of N protein in acidic phosphate buffers below pH 6.7 correlates with increased protonation of histidine residues.

Therefore, the choice of appropriate buffer and pH is particularly important when translating in vitro LLPS findings to the biologically relevant in cell behavior as viral infections are often accompanied by changes in intracellular pH.

## INTRODUCTION

**FIGURE 1:**
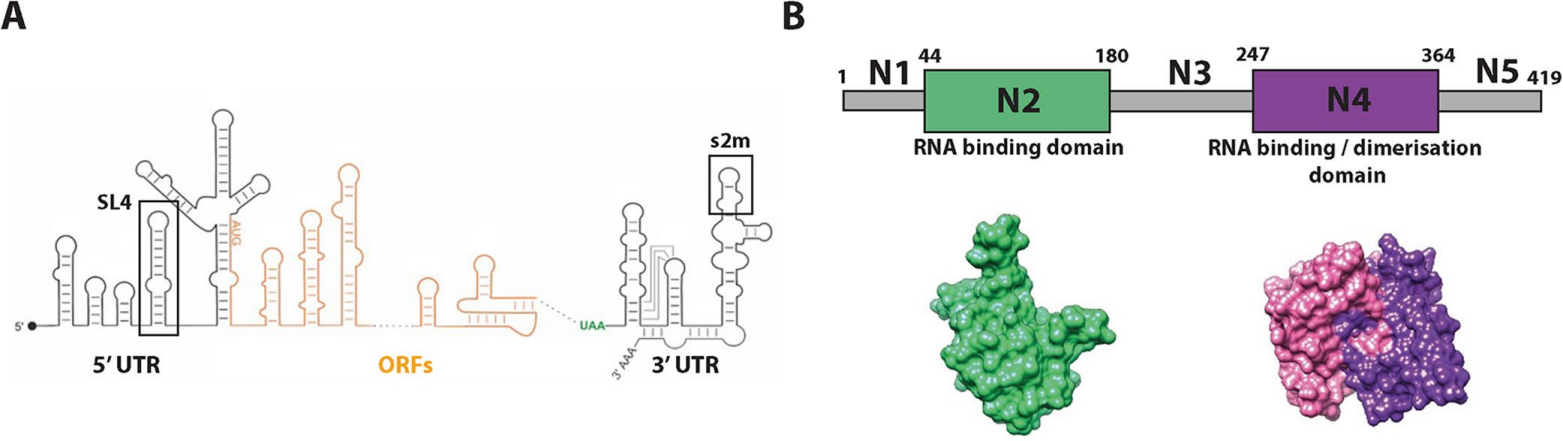
Schematic drawings of SARS-CoV-2 RNA genome and nucleocapsid (N) protein. (A) Schematic representation of the RNA secondary structures in the 5′ and 3′ untranslated regions (UTR) and open reading frames (ORF) of the SARS-CoV-2 genome. SL4 in the 5′UTR and the s2m in the 3′UTR are highlighted in the black boxes. A more detailed representation of both RNA stem loops can be found in Supplemental Figure S1. Adapted from (Wacker *et al.*, 2020). (B) Schematic drawing of the five domains in nucleocapsid protein. The N-terminal and C-terminal structured domains (N2 and N4, respectively) are coloured, and their predicted structures shown on the bottom. The grey parts of the protein sequence denote IDRs. Adapted from (Padroni *et al.*, 2023).

The COVID-19 pandemic is caused by the Severe Acute Respiratory Syndrome Coronavirus 2 (SARS-CoV-2; Wu *et al.*, 2020; Zhu *et al.*, 2020) and has resulted in almost seven million deaths (WORLD HEALTH ORGANISATION (WHO), 2023) worldwide as of November 2023. SARS-CoV-2 is an enveloped β-coronavirus with a 30′000 nucleotide long, single-stranded RNA genome, which possesses a 5′ cap and 3′ poly-A tail (Figure 1A; Finkel *et al.*, 2021). The SARS-CoV-2 genome encodes several proteins, among which the structural proteins Spike (S), Nucleocapsid (N), Envelope (E) and Membrane (M; Zhu *et al.*, 2020; Brant *et al.*, 2021; Finkel *et al.*, 2021). Many efforts have concentrated on the S protein due to its potential for vaccine development (Xia, 2021). More recently, research efforts are also undertaken to study the other three structural proteins in more detail, and especially the N protein.

N protein is a 46kDa RNA-binding protein with two structured domains flanked by three intrinsically disordered regions (IDR; Figure 1B; Bai *et al.*, 2021). The two structured domains, N2 and N4, are responsible for RNA-binding and dimerization of the protein (Zeng *et al.*, 2020; Bai *et al.*, 2021), respectively. Due to the high isoelectric point (pI) of N protein, it has a net positive charge at physiological pH (Zeng *et al.*, 2020). The main functions of N protein include virus replication through association with the viral replication-transcription complex (RTC; Savastano *et al.*, 2020; Bai *et al.*, 2021) as well as genome packaging through interaction with the virus genome (Chang *et al.*, 2014; Bai *et al.*, 2021; Cascarina and Ross, 2022). For the latter function, it is crucial that the virus can selectively find the viral genome among sub-genomic viral and host RNAs. However, the mechanism underlying this selection process is still unclear.

Liquid–liquid phase separation (LLPS) describes the demixing of two liquid phases which drives the formation of biomolecular condensates (Brocca *et al.*, 2020; Li *et al.*, 2022; Wei *et al.*, 2022). Reportedly, N protein undergoes phase separation in presence of RNA (Chang *et al.*, 2014; Savastano *et al.*, 2020; Jack *et al.*, 2021; Cascarina and Ross, 2022) and shows a preference for 5′ and 3′ genomic elements (Iserman *et al.*, 2020). To elucidate the phase separation behavior of N protein and its potential relation to genome viral packaging, it is essential to determine specific RNA elements that drive robust LLPS. In the present study, we concentrated our efforts on stem loop 4 (SL4) in the 5′UTR and the stem loop 2 motif (s2m) in the 3′UTR (both highlighted in Figure 1A; Supplemental Figure S1). SL4 has a profound effect on viral replication and plays an important role in directing subgenomic RNA synthesis (Yang *et al.*, 2011; Korn *et al.*, 2023; Vögele *et al.*, 2023). Furthermore, s2m is involved in regulating the virus life cycle (Gilbert and Tengs, 2021; Lulla *et al.*, 2021), and s2m mutations have been associated with increased viral fitness (Frye *et al.*, 2023), thus further strengthening the importance of this genomic element. Both RNAs were shown to interact with N protein (Imperatore *et al.*, 2022; Korn *et al.*, 2023), and we previously identified the binding interface of both N protein structured domains with s2m RNA (Padroni *et al.*, 2023).

In addition to the RNA sequence and structure, the formation of these membrane-less organelles depends on several factors, such as the concentration of the involved biomolecules, their sequence and structure (e.g., the presence of IDRs in proteins) or biophysical properties (e.g., net charge, local charge distribution), as well as external factors like temperature, pH, and ionic strength (Brocca *et al.*, 2020; Li *et al.*, 2022; Wei *et al.*, 2022). For example, phase separation of N protein with yeast RNA extract could be abrogated with salt concentrations above 300 mM NaCl, and was enhanced at pH 4.5 compared with pH 7.4 (Perdikari *et al.*, 2020).

In this study, we employed a variety of biochemical methods and NMR spectroscopy to investigate the effect of pH and the type of buffer (e.g., Tris, HEPES, or phosphate) on the phase separation of N protein with viral s2m and SL4 RNA elements. We demonstrate that both the buffer system and the pH can greatly influence LLPS of N protein, and that there is a substantial interplay between these two experimental factors. Further, we describe how the pH and buffer choices affect N protein on a molecular level and thereby alter its macroscopic LLPS behaviour.

## RESULTS

### LLPS of N protein with viral RNA is highly sensitive to pH

**FIGURE 2:**
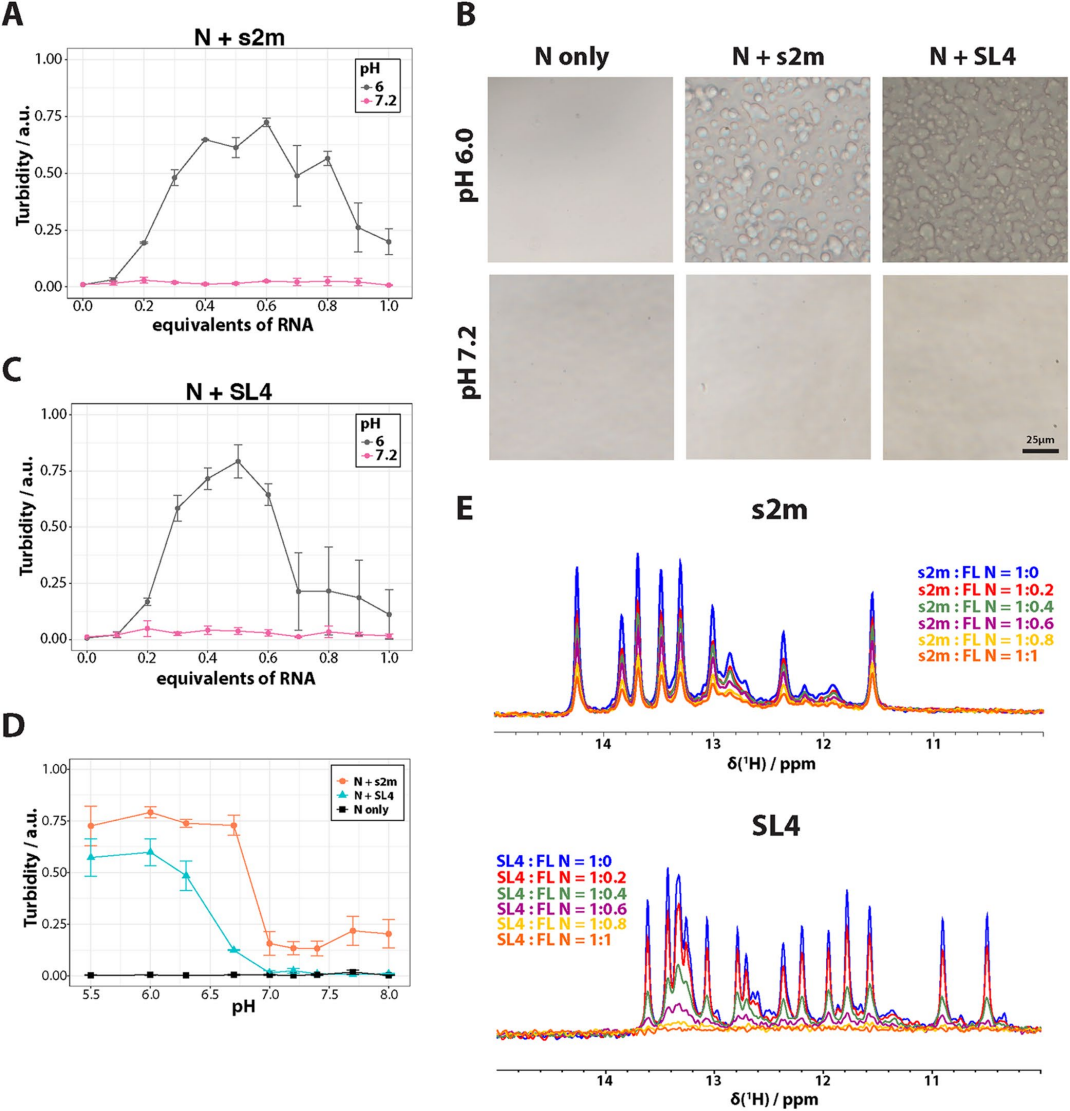
Phase separation of N protein with RNA in phosphate buffer is pH-dependent. (A) FL nucleocapsid (FL N) protein was prepared at 60 μM in P_i_ buffer, either at pH 6.0 or pH 7.2. Different molar equivalents of s2m RNA were added, and turbidity was measured as optical density at 600 nm (OD_600_). (B) Brightfield microscopy images of 60 μM FL N protein in P_i_ buffer, in presence or absence of 0.3 M equivalents s2m or SL4 RNA. Top: pH6.0, Bottom: pH7.2. Scale bar is 25 μm. Images are representative of *n* = 3 biological replicates. (C) Same experiment as in (A), but with SL4 RNA. (D) Turbidity measurements for the free protein as OD_600_ (black) and upon addition of 0.3 molar equivalents of s2m RNA (orange) or SL4 RNA (turquoise) with respect to different pH values of the P_i_ buffer. (E) 1D ^1^H SOFAST imino spectra of 100 μM s2m (top) or SL4 (bottom) RNA recorded in pH7.2 P_i_ buffer. FL N protein was titrated into the RNA samples in different molar equivalents as indicated in the legend. The buffer used in all experiments was 25 mM NaPi, 50 mM NaCl, pH as indicated. Data in (A), (C), and (D) represent mean ± standard error of the mean (SEM) of *n* = 3 biological replicates.

For our experiments, we used 25 mM sodium phosphate (P_i_) buffer with 50 mM NaCl at pH 7.2 to mimic the physiological conditions within a cell (Supplemental Figure S2). In contrast to the published literature (Iserman *et al.*, 2020; Jack *et al.*, 2021), we could not observe LLPS of N protein with ratios going from 0.1 to 1 molar equivalent of s2m or SL4 RNAs in pH 7.2 P_i_ buffer (Figure 2, A–C). However, when preparing the same samples P_i_ buffer at pH 6.0 for NMR experiments to slow down exchange of labile protons, we could detect the phase separation upon addition of both RNAs as demonstrated by turbidity and microscopy measurements (Figure 2, A–C), starting already at 0.2 equivalents of RNA.

This prompted us to investigate the influence of pH on the phase separation of N protein with RNA more closely by investigating a larger pH range (pH 5.5 – 8.0). Indeed, robust LLPS of N with s2m or SL4 RNA was observed at pH 6.7 and below, while phase separation was nearly abrogated at pH 7.0 and above (Figure 2D). Despite showing contrasting LLPS behavior, the two viral RNAs strongly interact with N protein at both pH 7.2 (Figure 2E) and pH 6.0 (Supplemental Figure S3) as can be concluded from the broadening of imino resonances in NMR titration experiments. These results suggest that LLPS behaviour of N protein with RNA is more dependent on pH than binding affinity.

### pH influences histidine protonation in N protein

**FIGURE 3:**
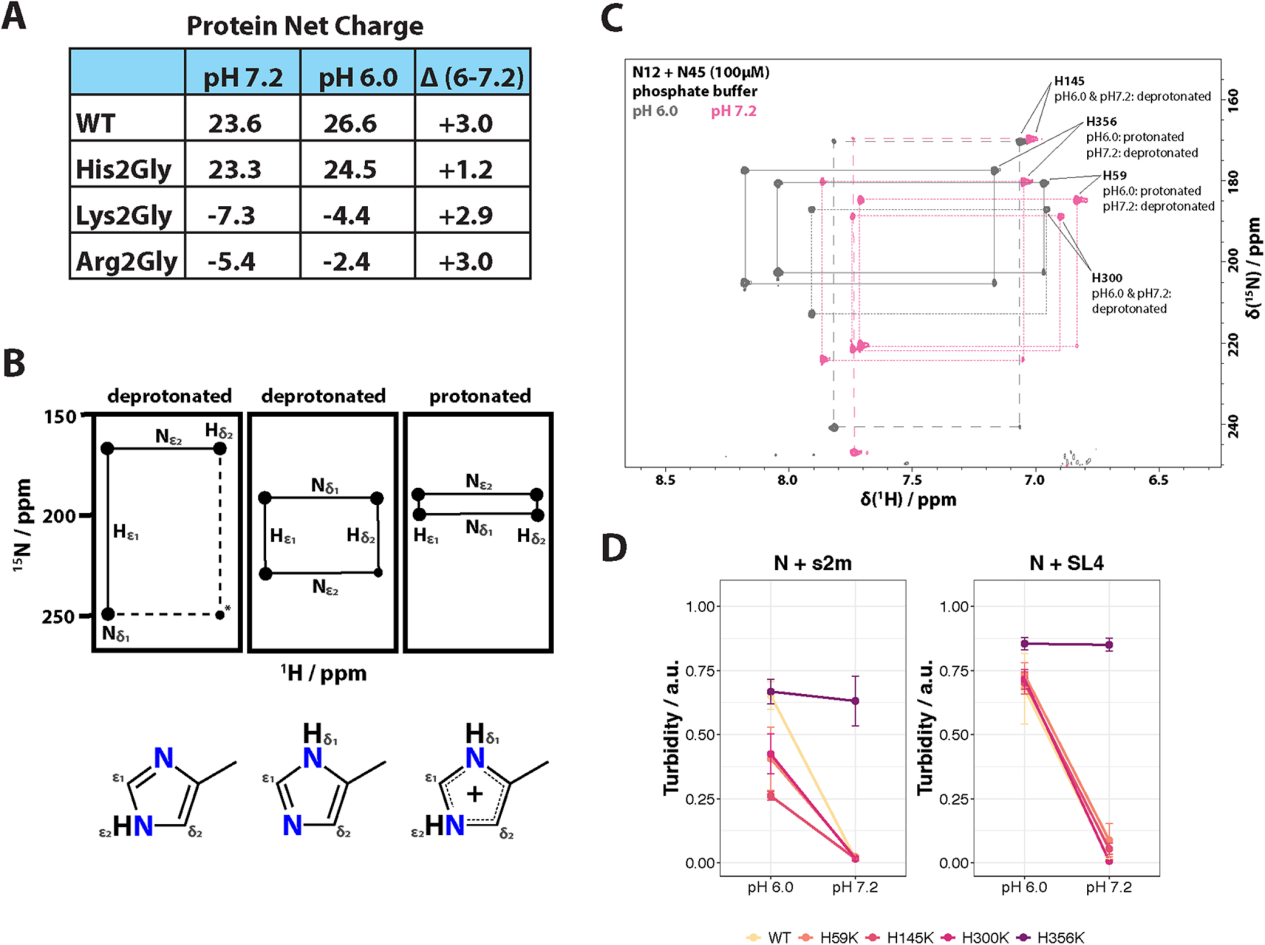
Protonation of residue H356 is crucial for LLPS in Pi buffer. (A) Theoretical predictions of protein net charge of wild-type (WT) FL N protein and three different mutants at pH 6.0 and 7.2. Calculations were done with ProtPi Software. (B) Top: Schematic drawing of two-dimensional NMR spectra of deprotonated (left, middle) and protonated (right) histidine residues. The asterisk (*) denotes peaks which are not always visible on the NMR spectra. The size of the peaks correlates to the relative signal strength of each cross-peak. Bottom: Structure of the deprotonated (left, middle) and protonated (right) imidazole ring of histidine residues. Adapted from (Simplaceanu *et al.*, 2000) (C) Two-dimensional ^15^N-^1^H HMQC spectra of 100 μM ^15^N-labeled N12 and N45 proteins in Pi buffer at pH 6.0 (grey) versus pH 7.2 (pink). The three or four peaks per histidine residue are connected as shown in (B). Solid lines indicate fully protonated histidine residues, dotted and dashed lines deprotonated histidine residues. Histidine residues were assigned using the Poisson-Boltzmann electrostatic surface simulation in Supplemental Figure S5 and by comparing the spectra of wild-type and H300K mutant protein. (D) Individual histidine (H) residues in FL N protein were mutated to lysine (K). Wild-type (WT) or H-to-K mutant N proteins were diluted in Pi buffer to 60 μM at pH 6.0 or 7.2. s2m (left) or SL4 (right) RNA was added (0.3 molar equivalents), and turbidity of the sample was measured as OD_600_. Data shows the mean ± SEM of *n* = 3.

The pH-dependence of N protein phase separation has been previously shown, though no explanation has been offered other than altered electrostatic interaction (Perdikari *et al.*, 2020; Zeng *et al.*, 2020; Cascarina and Ross, 2022). Because the pH-dependence of N protein phase separation in Figure 2D resembled a titration curve of positively charged amino acids, their protonation status may be crucial for LLPS. In silico calculations predicted a difference in protein net charge of +3 when changing the pH from 7.2 to 6.0. Yet, this difference was reduced to +1 charge when all histidines were mutated to glycine (His2Gly mutant). A similar change in net charge was not predicted for lysine-to-glycine (Lys2Gly) or arginine-to-glycine (Arg2Gly) mutants (Figure 3A).

Thus, we utilized NMR long-range HMQC experiments (Simplaceanu *et al.*, 2000) to observe histidine protonation in N protein at pH 6.0 and 7.2. Depending on the protonation state of the imidazole ring of histidine (i.e., one or two protons), ^15^N-^1^H correlation spectra reveal different numbers of the observed cross-peaks with distinct chemical shifts (Figure 3B). We found that two of the four histidines, H59 and H356, were fully protonated at pH 6.0 but deprotonated at 7.2 (Figure 3C; Supplemental Figures S, 4 and 5), which supports the theoretical predictions and might explain why LLPS of N protein is impaired at higher pH in P_i_ buffer. The fact that the two histidines undergoing protonation changes are located in the protein domains N2 and N4, respectively, further suggest that the two structured domains of N protein are involved in LLPS.

To test whether it is indeed charge that modulates phase separation, we replaced each histidine individually with lysine, giving rise to the following N protein mutants: H59K, H145K, H300K, and H356K. If deprotonation of histidine residues and thus the loss of charge is solely responsible for the loss of LLPS, our lysine mutants should phase separate with RNA at both pH values due to the high pI of this amino acid and subsequent protonation in both conditions. Indeed, turbidity measurements with these protein mutants in presence of s2m or SL4 RNA at both pH 6.0 and pH 7.2 showed that the H356K mutant phase separated with both RNAs at the two tested pH values (Figure 3D). In contrast, all other three mutants behaved like the wild-type protein and only underwent LLPS at pH 6.0 but not pH 7.2. This indicates that protonation of H356 specifically enabled N protein to phase separate in P_i_ buffer at physiological pH.

### The type of buffer strongly influences LLPS of N protein with viral RNA

However, this still didn’t explain the lack of LLPS with WT N protein at pH 7.2 although it had been reported in the literature. Thus, we further speculated that the type of buffer could also influence phase separation of N protein. By meticulously examining the experimental conditions under which LLPS of N protein with RNA was demonstrated in the literature, we found that Tris buffer was the prevalent choice for such experiments, followed by HEPES buffer, while P_i_ buffer was rarely used (Chang *et al.*, 2014; Iserman *et al.*, 2020; Savastano *et al.*, 2020; Jack *et al.*, 2021; Cascarina and Ross, 2022). Importantly, some studies even employed different buffer systems across experiments, but usually opted for Tris buffer when demonstrating LLPS by turbidity measurements or microscopy.

**FIGURE 4:**
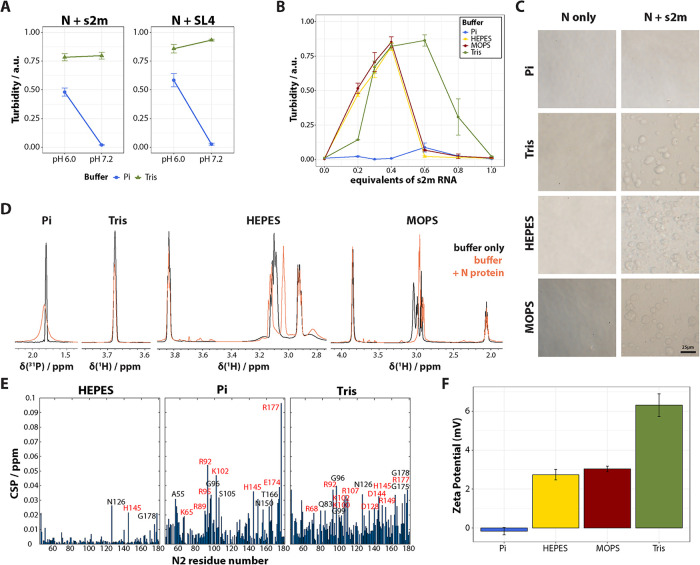
The ability of N protein to phase separate with RNA is influenced by the type of buffer molecule. (A) FL N protein was diluted in Pi or Tris buffer to 60 μM at pH 6.0 or 7.2. s2m (left) or SL4 (right) RNA was added (0.3 molar equivalents), and turbidity of the sample was measured as OD_600_, *n* = 3. (B) FL N protein was diluted to 60 μM in Tris, HEPES, MOPS or P_i_ buffer (pH 7.2). Different molar equivalents of s2m RNA were added, and turbidity was measured as OD_600_, *n* = 2. (C) Brightfield microscopy images of 60 μM FL N protein in the different buffers at pH7.2, in absence (left) or presence (right) of 0.3 molar equivalents s2m RNA. Scale bar is 25 μm. (D) 1D ^1^H or 1D ^31^P NMR spectra of 25 mM sodium phosphate, Tris, HEPES, or MOPS buffer, in absence (black) and presence of 100 μM ^15^N-labeled N12+N45 truncated proteins (orange). (E) Two-dimensional ^15^N-^1^H HSQC spectra of 100 μM ^15^N-labeled N12 + N45 were recorded in the four different pH 7.2 buffers. CSPs were calculated for all amino acids using the MOPS spectrum as reference. The CSP were plotted against the amino acid residues. Amino acid peaks that shifted significantly compared with the MOPS reference spectrum are indicated in the respective plot. Charged amino acids are written in red. Only the CSP of N2 domain are shown. (F) FL N protein was diluted in the respective buffers (25 mM buffer, no salt, pH 7.2) to 30-50 μM. Zeta potential of the samples was measured with the M3-PALS program on a Zetasizer Nano ZS, *n* = 2. Data in (A), (B), and (F) represent mean ± SEM.

Therefore, we tested additional buffers for our study. Initially we compared the LLPS behaviour of N protein with two viral RNAs at pH 6.0 and 7.2 in P_i_ and Tris buffer. Surprisingly, phase separation showed a very different behaviour in Tris buffer. While phase separation in P_i_ buffer is high at pH 6.0 and strongly reduced at pH 7.2 based on the sample turbidity, there is no change in phase separation in Tris buffer at either pH (Figure 4A). To rule out any protein batch effects, crossover experiments were performed. Essentially, the Tris protein stock solutions were diluted in P_i_ buffer for the turbidity measurements, and vice versa. N protein exhibited a phase separation behaviour akin to the mean of the LLPS behaviour in both buffers (Supplemental Figure S6). Moreover, patterns of histidine protonation were identical in Tris and P_i_ buffer at the respective pH values (Supplemental Figure S7). Together, these results suggest that pH only becomes relevant in the context of specific buffer systems (interaction effect).

To further strengthen our hypothesis that different buffers influence the ability to undergo LLPS, the turbidity experiments were repeated with s2m over a range of RNA equivalents at pH 7.2 in two additional buffer systems, HEPES and MOPS. We found that phase separation remained high in Tris buffer across a wider range of RNA concentrations compared with MOPS and HEPES buffer. In contrast, there was almost no LLPS in P_i_ buffer (Figure 4, B and C). Similar results were observed with SL4 RNA as well (Supplemental Figure S8). A similar effect has been described previously when examining the influence of different buffers on the phase stability of chicken egg-white lysozyme (Brudar and Hribar-Lee, 2021). The authors speculated that the interaction of buffer molecules with oppositely charged amino acids could affect the net charge of the protein and alter its phase stability (Brudar and Hribar-Lee, 2021).

### Electrostatic interactions of buffer molecules with N protein affect its net charge and ensuing phase separation behaviour

To investigate whether buffer molecules indeed interact with N protein and how they affect its net charge, we used NMR spectroscopy and electrophoretic light scattering. One-dimensional ^1^H spectra of HEPES, MOPS and TRIS buffer, and 1D ^31^P spectra of P_i_ buffer at pH 7.2 were recorded, first without protein and then in the presence of 100 μM N protein. Overlapping peaks of HEPES and MOPS buffers imply that N protein does not interact with mainly neutral HEPES and MOPS buffer molecules (Figure 4D). Being very sensitive to the pH, we attributed the shifts of some of the HEPES and MOPS resonances to slight pH differences throughout the samples. In contrast, there is a significant broadening and signal decay of Tris and P_i_ buffer signals, confirming an interaction of N protein with these charged buffer molecules (Figure 4D). Chemical shift perturbation (CSP) plots arising from ^15^N-^1^H HSQC NMR spectra acquired on N protein in the different pH 7.2 buffers demonstrated similar effects from the protein side (Figure 4E). There were more perturbed amino-acid residues compared with the spectra in HEPES and MOPS buffer. P_i_ and Tris buffer caused a significant increase in CSP of almost all residues, and especially the charged ones (highlighted in red). Phosphate predominantly affected positively charged amino acids, while Tris also perturbed aspartic acid (D) amide protons. Other perturbed residues labeled with black font in Figure 4E are uncharged but in very close proximity to the charged amino acids. Note that these differences emerge from the interactions with buffer molecules because the histidine protonation spectra were identical in all four buffer systems, confirming the same pH in the samples (Supplemental Figure S7).

Finally, the zeta (ζ) potential of free N protein in the different pH 7.2 buffers was measured to assess the correlation of protein net charge with the buffer-protein interaction. We found that the ζ-potential of N protein was positive in HEPES, MOPS, and Tris buffer, but near-neutral in P_i_ buffer (Figure 4F). Because ζ-potential is a proxy for the effective charge of a particle in solution (Bhattacharjee, 2016; Grisham and Nanda, 2020; Lunardi *et al.*, 2021), our results suggest that N protein is most positively charged in Tris buffer, slightly less positively charged in HEPES or MOPS buffer, and neutral in P_i_ buffer. Reduced protein net charge and thus reduced potential for electrostatic interactions could explain the lack of phase separation between N protein and viral RNA in P_i_ buffer, while LLPS was observable under the same conditions in the other tested buffers. Similarly, increased protein net charge would also explain why more equivalents of RNA were needed to induce reentrant behaviour (Banerjee *et al.*, 2017; Milin and Deniz, 2018) in Tris buffer compared with HEPES or MOPS buffer, as shown in Figure 4B.

## DISCUSSION AND CONCLUSION

In summary, we showed that increased protonation of histidine residues in N protein, which is caused by the acidification of the buffer, correlates with enhanced phase separation of N protein bound to viral RNA stem loops. Specifically, protonation of H356 seems to be crucial for LLPS of N protein in P_i_ buffer. As H356 is located in the N4 dimerization domain, its protonation leads to the addition of two positive charges in close proximity and thus increases local charge of the N protein. Apart from the changes in histidine protonation, local surface charge distribution of N protein is largely the same at pH 6.0 and pH 7.2, suggesting an important role of histidine residues for N protein phase separation upon pH change.

During viral infections, the intracellular pH reportedly decreases by as much as 0.4 units (Ciampor *et al.*, 1992), largely due to high rates of glycolysis (Allison and Sandelin, 1963; Singh *et al.*, 1974). Higher metabolic turnover during infection is necessary to produce large quantities of ATP and thus to support viral replication in the host cell (Allison and Sandelin, 1963; Singh *et al.*, 1974). While the resulting acidification of the cytoplasm can be at least partially compensated for by the host cell through increased H^+^ export (Gillies *et al.*, 1992), it might also serve to increase viral replication efficiency, for example, through facilitation of biomolecular condensate formation after histidine protonation. Similarly, lower pH of the cell culture medium favors the production of lentiviral vectors (Holic *et al.*, 2014), indicating an overall positive effect of slight acidification on virus replication.

Furthermore, our data also revealed an interplay between the pH and the choice of buffer system. In contrast to P_i_ buffer, no significant pH dependence was observed in Tris buffer. Instead, we showed that Tris and phosphate ions, which are positively and negatively charged at pH 7.2, respectively, interact electrostatically with charged amino acids of N protein and thereby affect the protein net charge. This leads to enhanced LLPS behaviour of N protein in Tris buffer and reduced phase separation ability in P_i_ buffer at physiological pH. It further seems that the alteration of protein net charge by buffer-protein interactions has a dominant effect on LLPS compared with histidine protonation upon pH changes. Histidine protonation was identical in all tested buffers and solely depended on the pH value, while the protein net charge and LLPS behaviour were drastically different between buffer systems. These results suggest that histidine protonation is less relevant for LLPS of N protein when the protein has a positive net charge in the first place (e.g., in Tris buffer), but the presence or absence of single charges becomes crucially important when the protein adopts an overall neutral charge (e.g., in Pi buffer).

Similar anion-protein interactions have been demonstrated for the positively charged proteins Antp homeodomain or BPTI, but importantly not for neutral ubiquitin protein (Yu *et al.*, 2021). Because an important interaction mode for LLPS are charge–charge interactions (Dignon *et al.*, 2020), it is no surprise that association of a protein with cations or anions (generally referred to as ion atmosphere [Lipfert *et al.*, 2014]) will alter protein net charge or local surface charge and thus influence LLPS behavior.

Nevertheless, the present findings also have important implications beyond the scope of this study. As multivalent electrostatic interactions play a significant role in phase separation of biological systems (Banerjee *et al.*, 2017; Mehta and Zhang, 2022), the choice of appropriate buffer and pH becomes crucial and should be taken into account. Moreover, while these parameters are usually considered individually, our data suggests that they must be considered in concert as well. These considerations are particularly important when translating in vitro LLPS findings to the biologically relevant *in cell* behaviour. Although the crowding agents, nucleotides, diverse cationic and anionic species, or small molecules present in a cell may all influence LLPS behaviour (Kilgore and Young, 2022), the considerations brought forward in this study provided mechanistic insights into phase separation of biological systems.

## MATERIALS AND METHODS

Request a protocol through *Bio-protocol*.

### Buffers

Unless stated otherwise, the experiment buffers used in this study were composed of 50 mM NaCl and 25 mM buffer molecule as indicated (i.e., sodium phosphate [NaPi], Tris, HEPES or MOPS). Buffers were adjusted to pH 6.0 or 7.2. Additionally, the phosphate buffers were adjusted to the following pH values: 5.5, 6.3, 6.7, 7.0, 7.4, 7.7, and 8.0.

### Protein expression

Sequences of the full-length (FL) nucleocapsid protein and the truncated N12 (N^1-180^), N234 (N^44-364^), and N45 (N^247-419^) proteins were inserted into pESPRIT vector (GenScript) in-frame with an N-terminal 6xHis-tag and under control of T7 promoter. The nucleocapsid protein constructs were expressed in *Escherichia coli* BL21 (DE3) overnight at 18°C after induction at an optical density of 0.6 with 1 mM isopropyl-β-D-1-thiogalactopyranoside (IPTG). Cells were harvested by centrifuging at 4′000 *g* and resuspended in [20 mM Tris pH 8.0, 1 M NaCl, 10 mM imidazole], lysed by a cell cracker, and centrifuged again at 35′000 *g* at 4°C. The supernatant was passed through Ni-NTA agarose resin (Qiagen) to immobilize the 6xHis-tagged proteins. Proteins were eluted with [20 mM Tris pH 8.0, 500 mM NaCl, 300 mM imidazole]. Samples were then dialyzed against [20 mM Tris pH 8.0, 300 mM NaCl, 5 mM β-mercaptoethanol] at 4°C overnight. Following TEV cleavage (in-house purified) and removal of the excess His-tag and TEV by Ni-affinity, samples were exchanged into the respective buffers with centrifugal concentrators (Vivaspin, MWCO 10kDa). ^15^N-labeled protein for NMR experiments was obtained by following the same procedure as outlined above, but bacteria were instead grown in M9 minimal medium supplemented with ^15^NH_4_Cl. All wild-type and mutant proteins expressed well, except for the mutant N12-H59K that expressed in the inclusion bodies and the small fraction that was purified from the lysate was highly aggregated.

### Site-directed protein mutagenesis

Site-directed mutagenesis N protein was carried out by PCR. To assess the effect of histidine residues on LLPS, we individually mutated histidine (H) to lysine (K) in FL N protein, giving rise to the following FL N mutants: H59K, H145K, H300K, and H356K. To assign the histidine NMR spectrum, we mutated one histidine residue each in N12 and N45 truncated proteins, resulting in the N12-H59K and N45-H300K mutants. Primers for mutagenesis were designed in a back-to-back orientation using the NEBaseChanger Tool. PCR with each primer-pair was carried out for 25 cycles using the HF Phusion DNA Polymerase (NEB) and 50 ng of the respective pESPRIT protein vector as template. Nonmutated plasmid templates were removed by DpnI treatment, and the linear double-stranded PCR products were circularized using kinase and ligase treatment, following the manufacturer’s instructions (NEB). Circularized mutated plasmids were transformed into chemically competent *E. coli* DH5α by heat-shock. Plasmids were amplified, miniprepped (Macherey-Nagel) and sent for sequencing to Microsynth, Switzerland.

### RNA in vitro transcription

RNAs were prepared from synthetic DNA templates containing the T7 promoter region. The first two nucleotides at the 5′ end of the template strand were modified to contain 2′O-Me groups. The respective DNA templates were annealed with a shorter, complementary DNA strand containing the T7 promoter region. These synthetic DNAs were purchased from Microsynth, Switzerland. The in vitro transcription reaction (6 mM rNTP, 30 mM MgCl_2_, in-house purified T7 polymerase) was incubated for at least 5 h at 37°C and supplemented with EDTA before anion exchange HPLC purification using a DNAPac-PA100 22 × 250 mm column heated to 85°C. After column equilibration and injection at 100% buffer A (6 M urea, 12.5 mM Tris–HCl, pH 7.4), a 0.25 min gradient to 15% buffer B (6 M urea, 12.5 mM Tris–HCl, pH 7.4, 0.5 M NaClO_4_) was used to elute excess of mononucleotides. The RNA was eluted using a slower gradient from 15 to 73% buffer B over 12.5 min, and the column was washed to 100% buffer B and reequilibrated to 100% buffer A before the following injection. The pure RNA fractions were identified by 16% PAGE containing 8M urea. RNAs were isolated by butanol extraction and lyophilized before resuspension into the respective buffers. Correct folding of the RNA constructs was ensured by heating to 95°C for 5 min and snap-cooling on ice before use.

### NMR spectroscopy

All NMR spectroscopy experiments were performed using Bruker AVNEO 500, 600, and 700 MHz spectrometers equipped with HCN or HCNP cryoprobes. 1D experiments were acquired in 5% D_2_O at 298K, using WATERGATE for water suppression. 1D ^1^H spectra were acquired with a spectral width of 22.3 ppm and centered at 4.7 ppm. 1D ^31^P spectra were acquired with a spectral width of 20.0 ppm and centered at 0 ppm. 1D ^1^H SOFAST experiments were centered on the imino region (12.5 ppm) and acquired with 120 ms interscan delay. Two-dimensional experiments were acquired in in 5% D_2_O at 303K. Two-dimensional ^15^N-^1^H HMQC spectra observing histidines were acquired with spectral widths of 13.6 ppm for ^1^H and 140 ppm for ^15^N, centered at 4.7 and 190 ppm, respectively. Two-dimensional ^15^N-^1^H HSQC spectra observing the backbone amines of the protein were acquired with spectral widths of 16.2 ppm for ^1^H and 35 ppm for ^15^N, centered at 4.7 and 117 ppm, respectively. The mixture of ^15^N-labeled N12 and N45 truncated proteins provided nicely resolved spectra and allowed the observation of N4 domain (which is otherwise NMR-invisible; Supplemental Figure S9), therefore it was used for all NMR experiments (unless stated otherwise). The same conclusions about histidine protonation were reached using the truncated protein N234, in which both structured domains are connected by a flexible linker (Supplemental Figure S10). Furthermore, the fold of N12+N45 or N234 truncated proteins was the same as in FL protein (Supplemental Figure S9). To assign the histidine residues in N4 domain, we recorded a two-dimensional ^15^N-^1^H HMQC spectra of ^15^N-labeled N45-H300K protein. Because N12-H59K did not express, we correlated changes in our NMR spectra to the surface charge simulations in Supplemental Figure S5 to assign the histidine residues in N2 domain. Before each NMR experiment, the pH of the sample was validated using a pH meter and readjusted if needed. Data were processed with Topspin 4.0.7 (Bruker). CSPs were calculated according to equation 1.




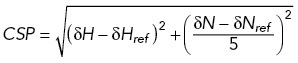




Equation 1. Calculation of CSPs of peaks in two-dimensional NMR spectra.

### Turbidity measurement

For the turbidity measurements, FL nucleocapsid protein was diluted in the respective buffers to a final concentration of 60 μM. RNA was added in the indicated molar ratios, mixed thoroughly by pipetting and measured immediately. Turbidity was measured by optical density at 600 nm (OD600) using a NanoDrop UV-Vis spectrophotometer (Thermo Fisher Scientific). Averaged turbidity values were obtained from measurement of multiple independent, freshly prepared samples (exact number of replicates indicated in respective figure legend). Data was visualized in R (version 4.2.1; R Core Team, 2022) as mean ± standard error of the mean (SEM).

### Brightfield microscopy

Samples for microscopy were prepared by diluting FL N protein in the respective buffers to a final concentration of 60 μM and adding RNA in the indicated molar ratios. Samples were mixed by pipetting and transferred to glass-bottom 384-well plates. Brightfield images were acquired within 5 min using a Sony α6400 APS-C DSLM camera body mounted to an Olympus CKX41 inverted microscope with a 40X objective.

### Zeta potential measurement

Zeta potential of the protein samples was measured with an electrophoretic light scattering apparatus (Zetasizer Nano ZS; Malvern Instruments) using folded capillary zeta cells (Malvern). The phase analysis light scattering programme (M3-PALS) was used to measure electrophoretic mobility of the samples. Thirty to fifty micromolar FL nucleocapsid protein (1.5–2.5 mg/ml) was measured in the indicated buffers (25 mM buffer ion, no salt, pH 7.2). Each measurement was done in technical triplicates. Zeta potential values were calculated from the electrophoretic mobility values using the Zetasizer software (Malvern). Data was visualized in R (version 4.2.1; R Core Team, 2022) as mean ± SEM.

## Supplementary Material



## Abbreviations used:

LLPS: liquid-liquid phase separation
N: Nucleocapsid
P _i_: inorganic phosphate
s2m: stem loop 2 motif
SL4: stem loop 4
