# Supplementary material for: Buffer choice and pH strongly influence phase separation of SARS-CoV-2 nucleocapsid with RNA
Source: Mol Biol Cell. 2024 Apr 19;35(5):ar73. doi: 10.1091/mbc.E23-12-0500 (PMC11151101; doi:10.1091/mbc.E23-12-0500)
Supplement: Supplementary file 1 [file mbc-35-ar73-s001.pdf]

Supplemental Materials

Molecular Biology of the Cell

Kathe *et al*.

Supplemental Material for

Buffer Choice and pH Strongly Influence Phase Separation of SARS-CoV-2 Nucleocapsid with RNA

Nina C. Kathe[1], Mihajlo Novakovic[1,*], Frédéric H.-T. Allain[1,*]

[1] *Institute of Biochemistry, ETH Zürich, Zürich, Switzerland.*

[*] *Corresponding authors. Email: mihajlo.novakovic@bc.biol.ethz.ch; allain@bc.biol.ethz.ch*

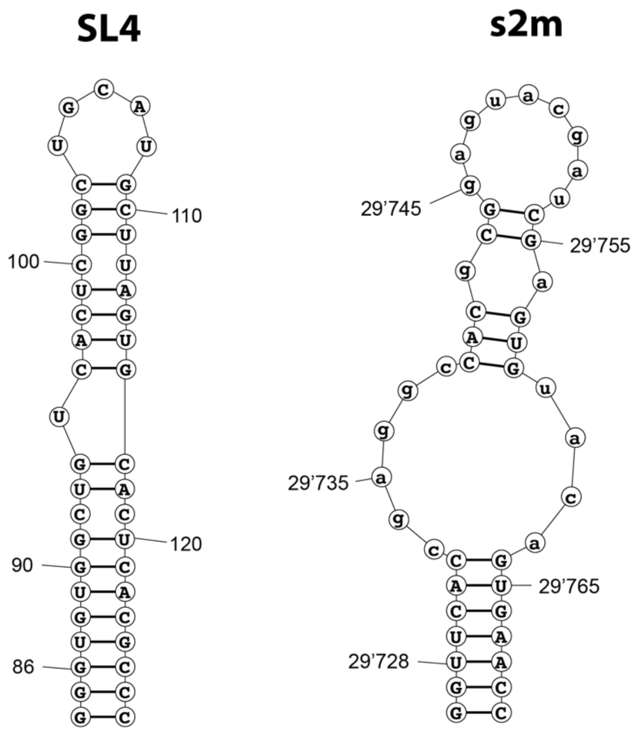

Supplemental Figure S1. Detailed structure and sequence of SL4 and s2m RNA elements. Note that two GC pairs were added at the bottom of the stem to make a more stable stem loop.

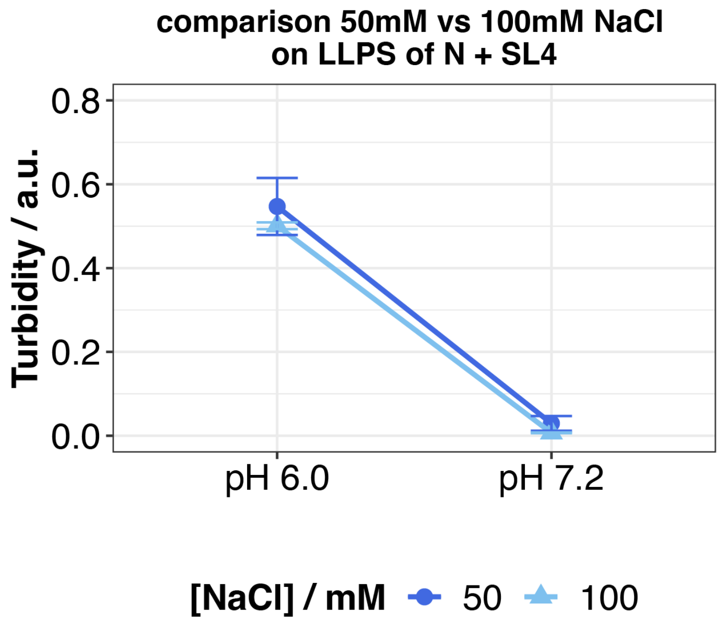

Supplemental Figure S2. Effect of 50mM vs 100mM NaCl on LLPS behaviour of N protein with SL4 RNA. To compare if our buffers with 50mM NaCl result in the same LLPS behaviour than we would get inside a cell (osmolarity approx. 100mM salt), FL N protein stocks were diluted to 60µM in Pi buffer containing either 50mM or 100mM NaCl. SL4 RNA was added (0.3 molar equivalents), and turbidity of the samples was measured as OD600. Both pH 6.0 and pH 7.2 were tested. Data shows mean ± SEM of n=2 biological replicates.

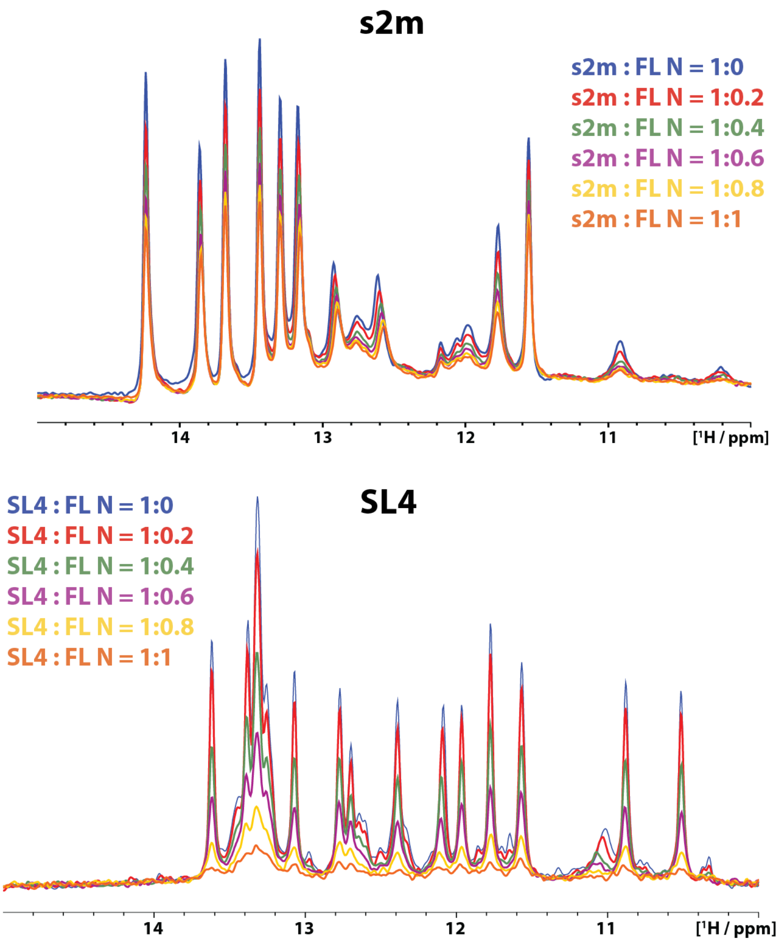

Supplemental Figure S3. 1D ^{1}H SOFAST of s2m and SL4 RNA in presence and absence of FL N protein at pH 6.0. 1D ^{1}H SOFAST spectra were recorded of 100µM s2m (top) or SL4 (bottom) RNA in pH6.0 P_i buffer (25mM NaPi, 50mM NaCl, pH6.0). FL N protein was titrated into the RNA samples in different molar equivalents as indicated.

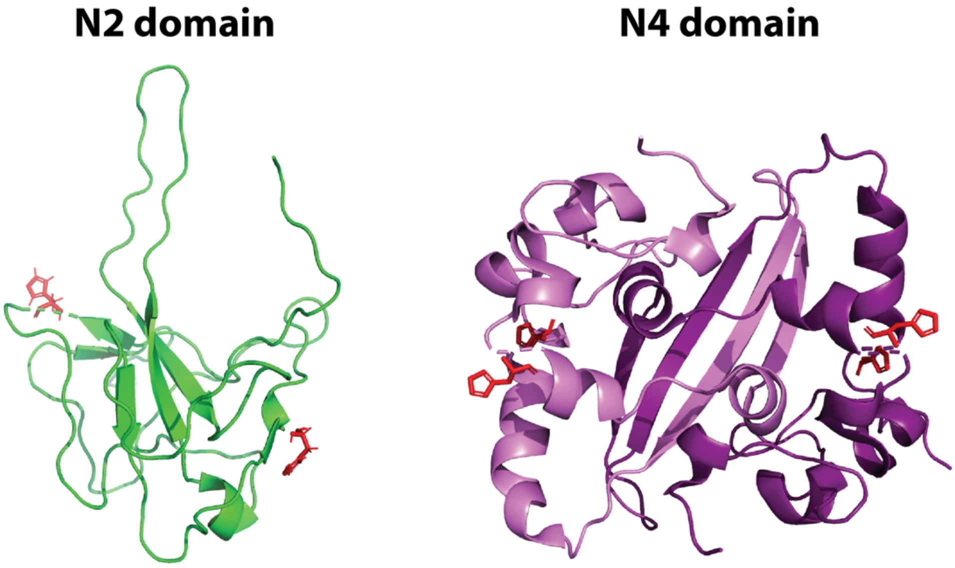

Supplemental Figure S4. Position of histidine residues in N2 and N4 domains of FL N protein. N2 (left; PDB 6YI3) and N4 (right; PDB 6YUN) domains of N protein were visualised in PyMOL in

green and purple, respectively. The four histidine residues of the protein are highlighted in red. Note that N4 domain is shown as homodimer (shaded in light and dark purple), hence four histidines are apparently visible in N4 domain.

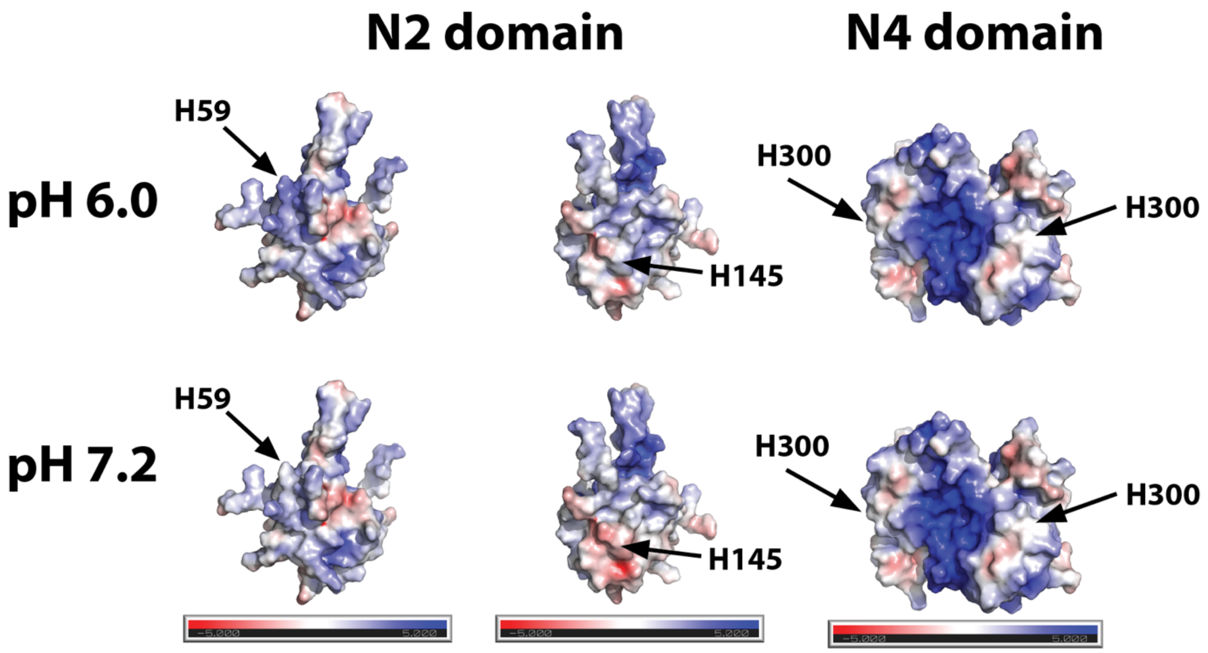

Supplemental Figure S5. Surface charge distribution in N2 and N4 domains of FL N protein at pH6.0 and pH7.2. Structures of N2 domain (PDB: 6YI3) and N4 domain (PDB: 7DE1) were prepared using the PDB2PQR algorithm (Jurrus et al., 2018), and electrostatic properties were simulated at pH6.0 (top) and pH7.2 (bottom) using APBS (Adaptive Poisson-Boltzmann Solver). Surface charge distributions were visualised in PyMOL. The positions of histidine residues are indicated with arrows. Note that H356 is buried inside the structure. Also note that N4 is depicted as homodimer.

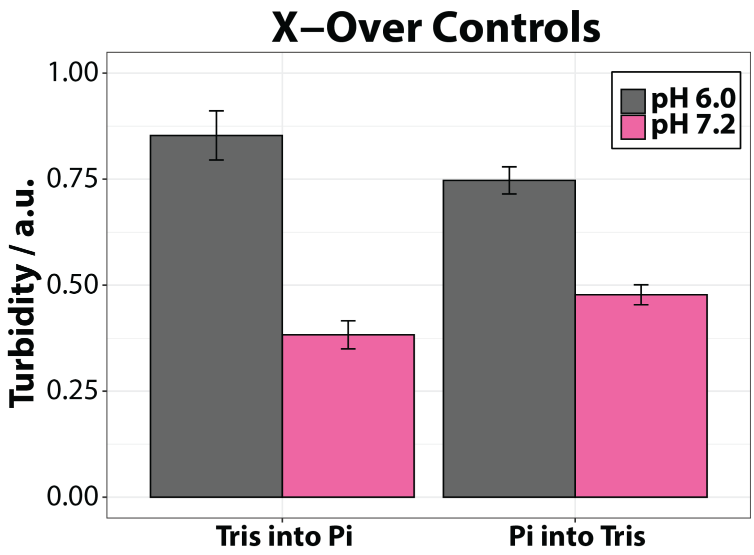

Supplemental Figure S6. Cross-Over Experiments. FL N protein stocks in Tris buffer were diluted to 60μM in Pi buffer and vice-versa. S2m RNA was added (0.3 molar equivalents), and turbidity of the samples was measured as OD_{600}. Both pH 6.0 and pH 7.2 were tested. N=2. Bars show mean ± SEM.

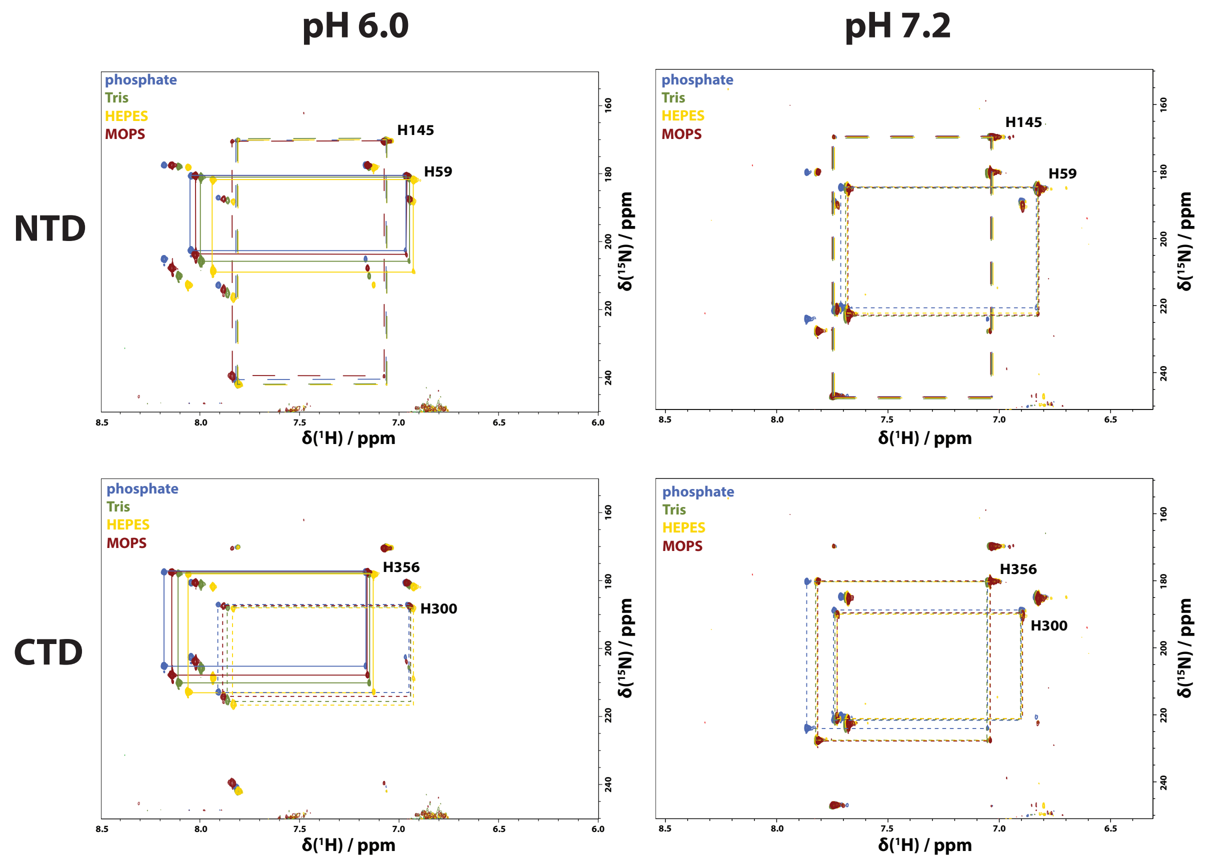

Supplemental Figure S7. Histidine protonation of N protein in four different buffers at pH7.2. 2D ^{15}N-^{1}H HMQC spectra of 100µM ^{15}N-labelled N12 and N45 proteins in the four different buffers at pH 6.0 (left) vs pH 7.2 (right). The peaks of each histidine residue are connected as shown in Figure 3B. Solid lines indicate fully protonated, dotted and dashed lines deprotonated histidine residues. For clarity, the lines were drawn for the two histidines in N2 domain in the top row, and for the histidines in N4 domain in the bottom panels.

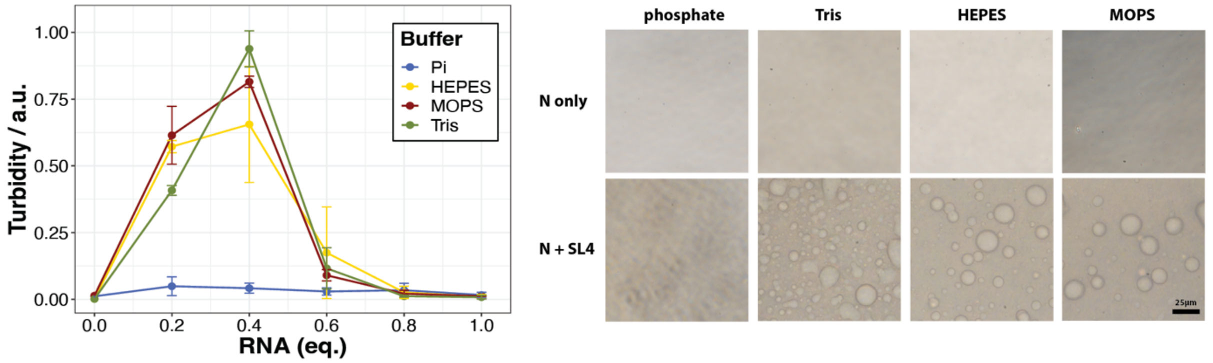

Supplemental Figure S8. LLPS of N protein with SL4 RNA in different buffers at pH7.2. Left: FL N protein was diluted to 60µM in Tris, HEPES, MOPS or Pi buffer (25mM buffer ion, 50mM NaCl, pH 7.2). Different molar equivalents of SL4 RNA were added, and turbidity was measured as OD600. Data shows mean ± SEM of n=2. Right: Brightfield microscopy images of 60µM FL N protein in the different buffers at pH7.2, in absence (top) or presence (bottom) of 0.3 molar equivalents SL4 RNA. Scale bar is 25µm.

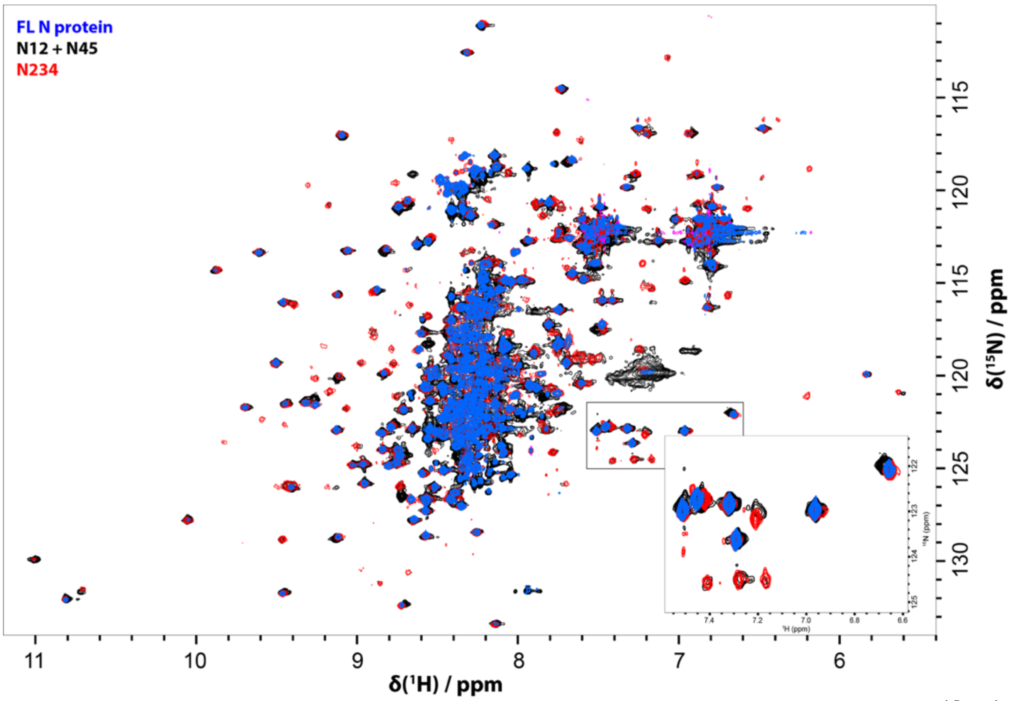

Supplemental Figure S9. Overlay of FL N, N12+N45 and N234 protein spectra. 2D ^{15}N-^{1}H HSQC spectra were recorded of full-length N protein (FL N, blue), the mixture of N12+N45 proteins (black) and N234 protein (red). All spectra were recorded with 100µM protein in pH6.0 phosphate buffer. The inset shows some peaks from N2 and N4 domains in more detail. Note that peaks from N4 domain are only visible in N234 and N12+N45 truncated proteins, but not FL N protein.

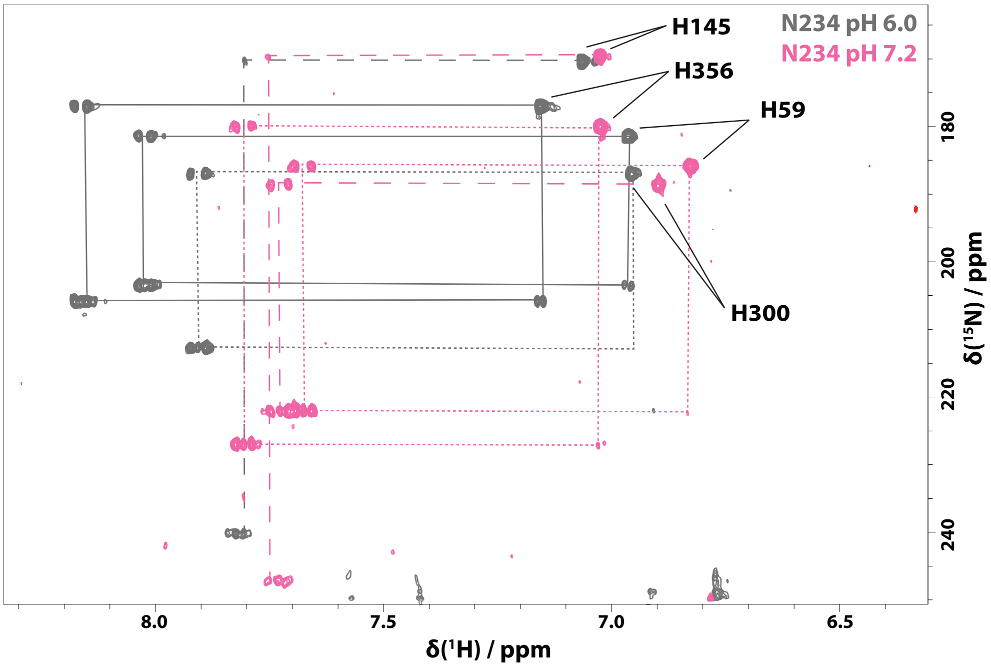

Supplemental Figure S10. Histidine protonation of N234 protein in pH 6.0 and pH 7.2 Pi buffer. 2D ^{15}N-^{1}H HMQC spectra of 100µM ^{15}N-labelled N234 proteins in the phosphate buffers at pH 6.0 (grey) vs pH 7.2 (pink). The peaks of each histidine residue are connected as shown in Figure 3B. Solid lines indicate fully protonated, dotted lines partially protonated and dashed lines deprotonated histidine residues.
