# Supplementary material for: Buffer choice and pH strongly influence phase separation of SARS-CoV-2 nucleocapsid with RNA
Source: Mol Biol Cell. 2024 Apr 19;35(5):ar73. doi: 10.1091/mbc.E23-12-0500 (PMC11151101; doi:10.1091/mbc.E23-12-0500)
Supplement: Supplementary file 2 [file reviewer_reportE23-12-0500.pdf]

Buffer choice and pH strongly influence phase separation of SARS-CoV-2 Nucleocapsid with RNA

Nina Kathe, Mihajlo Novakovic, and Frédéric Allain

Corresponding author(s): Frédéric Allain, ETH Zurich

Review Timeline:

Submission Date:	2023-12-22
Editorial Decision:	2024-02-07
Revision Received:	2024-03-18
Accepted:	2024-03-20

Editor-in-Chief: Matthew Welch

Transaction Report:

(Note: With the exception of the correction of typographical or spelling errors that could be a source of ambiguity, letters and reports are not edited. The original formatting of letters and referee reports may not be reflected in this compilation.)

RE: Manuscript #E23-12-0500
TITLE: "Buffer choice and pH strongly influence phase separation of SARS-CoV-2 Nucleocapsid with RNA"

Dear Dr. Allain:

We have received reviews from two experts in the fields of phase separation and the chemical properties of the cytoplasm. The reviewers were enthusiastic about the approach and conclusions of the study, and they agree that the findings will benefit the field of biomolecular condensates. Thus, both are in favor of publication, contingent on a few minor experiments and some rephrasing and expansion of the conclusions.

At minimum, the reviewer's revisions should include:
1. An experimental test of the importance of the two Histidines in the N protein to phase separation. The authors suggest that a His2Gly mutation should affect how protein net charge changes from pH 6.0 to 7.2. This should be tested with a turbidity assay, as done for the WT protein in Figure 2 (reviewer 1).

2.Turbidity and microscopy data of N + SL4 in Figure 4, panels B and C (reviewer 1).

3.Reassessment of the authors' statement about what constitutes a physiological buffer (reviewer 2).

4.Expansion of the discussion to talk about how the current results help us understand viral infection and replication in a complex cellular environment (both reviewers).

Sincerely,
Jeffrey Woodruff
Monitoring Editor
Molecular Biology of the Cell

Dear Dr. Allain,

The review of your manuscript, referenced above, is now complete. The Monitoring Editor has decided that your manuscript requires minor revisions before it can be published in Molecular Biology of the Cell, as described in the Monitoring Editor's decision letter above and the reviewer comments (if any) below.

A reminder: Please do not contact the Monitoring Editor directly regarding your manuscript. If you have any questions regarding the review process or the decision, please contact the MBoC Editorial Office (mboc@ascb.org).

When submitting your revision include a rebuttal letter that details, point-by-point, how the Monitoring Editor's and reviewers' comments have been addressed. (The file type for this letter must be "rebuttal letter"; do not include your response to the Monitoring Editor and reviewers in a "cover letter.") Please bear in mind that your rebuttal letter will be published with your paper if it is accepted, unless you have opted out of publishing the review history.

Authors are allowed 15 days to submit a revision. If this time period is inadequate, please contact us immediately at mboc@ascb.org.

In preparing your revised manuscript, please follow the instruction in the Information for Authors (www.molbiolcell.org/info-for-authors). In particular, to prepare for the possible acceptance of your revised manuscript, submit final, publication-quality figures with your revision as described.

To submit the rebuttal letter, revised version, and figures, please use this link (please enable cookies, or cut and paste URL):
Link Not Available

MBoC offers the option to publish your work with immediate open access. Open access can increase the discoverability and usability of your research. If you would like to publish your paper with immediate open access but did not select this option during initial submission, please contact us at mbc@ascb.org..

Authors of Articles and Brief Communications whose manuscripts have returned for minor revision ("revise only") are encouraged to create a short video abstract to accompany their article when it is published. These video abstracts, known as Science Sketches, are up to 2 minutes long and will be published on YouTube and then embedded in the article abstract.

Science Sketch Editors on the MBoC Editorial Board will provide guidance as you prepare your video. Information about how to prepare and submit a video abstract is available at www.molbiolcell.org/science-sketches. Please contact mboc@ascb.org if you are interested in creating a Science Sketch.

Thank you for submitting your manuscript to MBoC. Please do not hesitate to contact this office if you have any questions.

Sincerely,

Eric Baker
Journal Production Manager
MBoC Editorial Office
mbc@ascb.org

Reviewer #1 (Remarks to the Author):

In the manuscript entitled "Buffer choice and pH strongly influence phase separation of SARS-CoV-2 nucleocapsid with RNA," by Kathe and colleagues, the authors investigate the role of pH and type of buffer in the phase separation of nucleocapsid protein (N) with structured RNA fragments, SL4 and s2m, from SARS-CoV-2 RNA. The authors initially observe that N + s2m and N + SL4 undergo phase separation at pH 6.0 but not at pH 7.2. Titration of pH with N + s2m shows a sharp decrease in condensation between pH 6.7 and 7.0. NMR analysis of N demonstrates that two of four surface histidine residues on domains of N are protonated at pH 6.0 but not at pH 7.2; the authors posit that this increase in net charge of N promotes condensation with RNA. The authors then investigate the role of the type of buffer in phase separation of N and RNA. They find that in the presence of Pi buffer, s2m + N readily undergo phase separation at pH 6.0 but not at pH 7.2 while in the presence of Tris buffer, s2m + N undergo phase separation regardless of pH. The authors repeated these experiments using HEPES and MOPS, two common buffers used in in vitro phase separation studies, and found that s2m + N undergoes phase separation at pH 6.0 and pH 7.2, although the extent to which they condense seems to depend on the chosen buffer. The results reported here are important for a wide swath of condensate research that depends on in vitro reconstitution and offer unique insight into why different buffers can influence condensation.

Overall, this study provides clear evidence of the importance of pH for the phase separation of N with RNA fragments and the mechanism by which modulation occurs. The use of NMR to investigate histidine protonation and buffer-residue interactions provides the phase separation community with molecular insights into how pH and buffer selection can influence phase separation. However, the current manuscript would be bolstered by a few additional experiments and a more extensive discussion of how these results provide insights into what may happen in the complex cellular environment. If the concerns outlined below can be adequately addressed, this reviewer will support the publication of this manuscript in Molecular Biology of the Cell.

Concerns:
1) A major concern this reviewer has is as follows: additional experiments should be done to parallel data reported in Figures 1B and D and Figures 4B and C. Figure 1B should include an experiment in which N + s2m + SL4 is mixed at pH 6.0 and pH 7.2. This experiment would be a better mimic of the physiological situation in which s2m and SL4 likely coexist at equimolar concentrations on a single strand of RNA and provide the reader with context for N and RNA phase behavior when both structural elements are present. Figure 1D should also include a turbidity assay for N + SL4 as pH is titrated up. It will be interesting to see if the behavior of the system with SL4 diverges from s2m. And again, in Figure 4, s2m and SL4 are included in Figure 4A, so SL4 should be included in Figures 4B and 4C. Breaking down the contributions of s2m and SL4 is essential, but it raises questions of how each contribute to phase separation in tandem. It seems that the authors are in a good position to address this.
2) A helpful experiment related to point 1 above would be to test whether it is indeed charge that modulates phase separation. While the theoretical predictions in Figure 3A are helpful, the authors could use this as the basis for experimental probing of the effects of charge. The authors could probe the importance of each histidine by replacing them with lysine or arginine individually or in combination. By probing different combinations of positive charge on the surface of the domain, the authors can further probe N phase separation and deepen the understanding of specific surface charge contribution.
3) In the second line of the introduction, should "Mio" be "million?"
4) In the fourth and fifth line of the second paragraph of the introduction, please state that physiological pH is 7.2 to reduce potential confusion for the reader later in the manuscript.
5) In the fourth paragraph of the introduction, biophysical properties (e.g., the presence of IDRs in proteins), isn't as clear as it could be. IDRs are features of proteins while biophysical properties would include net charge, charge distribution, hydrophobic blocks, etc. Instead of presence of IDRs, the authors may want to state that net and local charge can be important biophysical characteristics of IDRs or domain surfaces. Doing so will lead more directly into their finding that the protonation of histidine residues is important for phase separation of N with RNA.

6) In the final paragraph of the introduction, please include the types of buffers used in the study so that the reader is aware of what the authors mean by "types of buffers." As written, this statement is too vague.
7) In Supplemental Figure S3, the position of the His residues on the surface of the domains is helpful. This figure could be made more impactful by including surface charge density with and without protonation of the His residues so that the reader can readily visualize how altering the charge of the His residues alters the local surface charge landscape.
8) In the final paragraph of the results section "The type of buffer strongly influences LLPS of N protein with viral RNA" is stated. What is meant by "most intense" when referring to Tris-related phase separation? Is it that turbidity remains higher for Tris across a wider range of s2m concentrations? Or did the authors have something else in mind. When looking at the images in Figure 4C, it might be that phase separation occurs as readily in HEPES, perhaps even more. Clearly defining what is meant by "most intense" will be helpful.
9) The final sentence of the results section states that "increased protein net charge..." alter phase behavior. This can be true, but another possibility should also be considered. Could it also be that increased local surface charge also can play a role? A charge map of the domains where the Histidine residues reside will be helpful for this point. If local charge may be important, perhaps the authors can also discuss this possibility.
10) The discussion should be expanded to include a more detailed discussion of how these results relate to the complex cellular environment. In the third paragraph, the authors state that the intracellular pH can drop by as much as 0.4 units upon viral infection. This would mean that the pH drops from 7.2 to 6.8, which is still too high for phase separation of N + s2m based on Figure 1D. However, it could be that the crowding of the cell or general "buffer" characteristics of the nucleotides, proteins, salts, small molecules, etc. in the cytoplasm may promote N phase separation. It may also be that inclusion of SL4 would induce phase separation at this pH (see points 1 and 2 above for potential experiments to include). A more detailed discussion would provide the reader with a more complete picture of how this study contributes to the greater discussion of the relevance of in vitro assays to cellular behaviors.

Reviewer #2 (Remarks to the Author):

Understanding the molecular basis and physical rules of biomolecular condensation is currently the subject of extensive efforts in the community. Specifically, condensation is known to be affected by multiple variables, for instance salt concentration, pH, temperature, buffer composition, but untangling who does what between these different variables is not always obvious, precluding our understanding of this important biological phenomenon. In this study, Kathe and colleagues investigate this question in vitro using the Nucleocapsid (N) protein from SARC-CoV-2 as a model system and a variety of biochemical assays, including NMR.

Specifically, the authors shows that phase separation of N protein with RNA is strongly pH dependent, with a sharp decrease of condensation when pH gets higher than 6.7 in phosphate buffer. This does not depend on the ability of the protein to bind the RNA, implying that RNA binding does not necessarily correlates with the phase separated behaviour. Histidines are known to change charge around these pH values, and the authors indeed show experimentally that two histidines of the N protein get protonated when pH drops and thus change charge, therefore providing a rationale for the pH sensitivity of the phase separation behaviour. Then the authors make a very nice observation that there is an interplay between the pH and the buffer used to establish this pH with regard to phase separation. Indeed, they found that while a shift from pH 6 to 7.2 strongly inhibits phase separation in Phosphate buffer, it does not occur in Tris, MOPS or Hepes buffers, all robustly supporting phase separation at pH 7.2. As histidine protonation is the same at a given pH between the different buffers, this suggested an interaction effect between the buffer and the nucleocapsid protein. They then show experimentally that Tris and Phosphate buffers strongly interact with the N protein by NMR. They also measured the Zeta potential of the protein in the different buffers, which showed a change of the effective charge of the protein (most positively charged in Tris buffer compared to neutral in phosphate buffer, all at a fixed pH of 7.2).

I find particularly interesting that given a constant pH and salt concentration, the choice of the specific molecule used to buffer the solution profoundly impacts the ability of the SARS-CoV-2 nucleocapsid to phase separate, This of course makes perfect sense with known biochemistry given the effective charge of the buffer molecule, which can (or not) shield the charged residues at the surface of the nucleocapsid. But I think it's a very nice example where the buffer choice matters and should help the community interpreting/designing their experiments. Figures are nicely rendered and methods are adequate in my opinion. I recommend publication, provided the author address my (minor) comment bellow.

My main comment about this paper is that I don't think the buffer the authors used can "to mimic the physiological conditions within a cell", page 3. Their buffer only has 50 mM NaCl and so has an osmolarity that is twice less than that of a cell. I think this matters, as salt concentration is also known to affect phase separation (plus the whole paper is about charges). Obviously, I'm not suggesting the authors redo all their experiments with a higher salt concentration (the set of experiments presented is all coherent and I think support the claims of the paper). But if the authors want to keep that specific sentence, they should at least do one control showing that a physiological salt concentration does not affect the ability of the N protein to phase separate in the presence s2m upon pH change. I think keeping this relation to physiological conditions is important, especially because the authors make a very nice point in discussion that intracellular pH drops upon infection, which could favour phase separation, which I think is a cool idea (it might be related to some reports that lowering cell culture pH improves the production of lentiviral

and retroviral vectors, Holic et al. Human gene therapy clinical development, 2014).

Eidgenössische Technische Hochschule Zürich
Ecole polytechnique fédérale de Zurich
Politecnico federale di Zurigo
Swiss Federal Institute of Technology Zurich

18/03/2024

Institute of Biochemistry
Prof. Frédéric Allain

Phone +41 44 633 39 40
E-mail allain@bc.biol.ethz.ch
Address Hönggerbergring 64
8093 Zürich
Switzerland

Manuscript ID: #E23-12-0500

Buffer choice and pH strongly influence phase separation of SARS-CoV-2 Nucleocapsid with RNA

Nina C. Kathe[1], Mihajlo Novakovic[1*], Frédéric H.-T. Allain[1*]

[1]Department of Biology, Institute of Biochemistry, ETH Zurich, Zurich, Switzerland
*Corresponding authors. Email: mihajlo.novakovic@bc.biol.ethz.ch, allain@bc.biol.ethz.ch

Response letter

Dear Mr. Woodruff,

Thank you for your email of 7th February 2024 containing the constructive comments of the referees on our manuscript "**Buffer choice and pH strongly influence phase separation of SARS-CoV-2 Nucleocapsid with RNA**". As can be appreciated from our responses below, we have now prepared a revised manuscript addressing all the critiques and suggestions raised by the reviewers. We provided our responses in blue font. For completion we are also submitting a "highlighted" version of the manuscript marking all the changes we made to the text, together with the "clean" version of the paper's main text and the SI.

Reviewer: 1

In the manuscript entitled "Buffer choice and pH strongly influence phase separation of SARS-CoV-2 nucleocapsid with RNA," by Kathe and colleagues, the authors investigate the role of pH and type of buffer in the phase separation of nucleocapsid protein (N) with structured RNA fragments, SL4 and s2m, from SARS-CoV-2 RNA. The authors initially observe that N + s2m and N + SL4 undergo phase separation at pH 6.0 but not at pH 7.2. Titration of pH with N + s2m shows a sharp decrease in condensation between pH 6.7 and 7.0. NMR analysis of N demonstrates that two of four surface histidine residues on domains of N are protonated at pH 6.0 but not at pH 7.2; the authors posit that this increase in net charge of N promotes condensation with RNA. The authors then investigate the role of the type of buffer in phase separation of N and RNA. They find that in the presence of Pi buffer, s2m + N readily undergo phase separation at pH 6.0 but not at pH 7.2 while in the presence of Tris buffer, s2m + N undergo phase separation regardless of pH. The authors repeated these experiments using

HEPES and MOPS, two common buffers used in in vitro phase separation studies, and found that s2m + N undergoes phase separation at pH 6.0 and pH 7.2, although the extent to which they condense seems to depend on the chosen buffer. The results reported here are important for a wide swath of condensate research that depends on in vitro reconstitution and offer unique insight into why different buffers can influence condensation.

Overall, this study provides clear evidence of the importance of pH for the phase separation of N with RNA fragments and the mechanism by which modulation occurs. The use of NMR to investigate histidine protonation and buffer-residue interactions provides the phase separation community with molecular insights into how pH and buffer selection can influence phase separation. However, the current manuscript would be bolstered by a few additional experiments and a more extensive discussion of how these results provide insights into what may happen in the complex cellular environment. If the concerns outlined below can be adequately addressed, this reviewer will support the publication of this manuscript in Molecular Biology of the Cell.

We are grateful for the positive comments, and we try to address all the points below.

Concerns:

1) A major concern this reviewer has is as follows: additional experiments should be done to parallel data reported in Figures 1B and D and Figures 4B and C. Figure 1B should include an experiment in which N + s2m + SL4 is mixed at pH 6.0 and pH 7.2. This experiment would be a better mimic of the physiological situation in which s2m and SL4 likely coexist at equimolar concentrations on a single strand of RNA and provide the reader with context for N and RNA phase behavior when both structural elements are present. Figure 1D should also include a turbidity assay for N + SL4 as pH is titrated up. It will be interesting to see if the behavior of the system with SL4 diverges from s2m. And again, in Figure 4, s2m and SL4 are included in Figure 4A, so SL4 should be included in Figures 4B and 4C. Breaking down the contributions of s2m and SL4 is essential, but it raises questions of how each contribute to phase separation in tandem. It seems that the authors are in a good position to address this.

Regarding Figure 2B (we believe the reviewer meant Figures 2B and 2D, and not Figure 1B,D), we originally tested both RNAs separately to get a better picture of the protein's behaviour in presence of viral RNA. However, following the reviewer's suggestion, we mixed N protein with both s2m and SL4 RNAs (0.15 eq. RNA each to keep total RNA the same) in phosphate (Pi) buffer at both pH 6.0 and pH 7.2. The samples were imaged using a brightfield microscope and are shown below. Droplets are also formed when both RNAs are added simultaneously, but only in pH6.0 Pi buffer and not in pH7.2 Pi buffer, as is the case also with both RNAs individually.

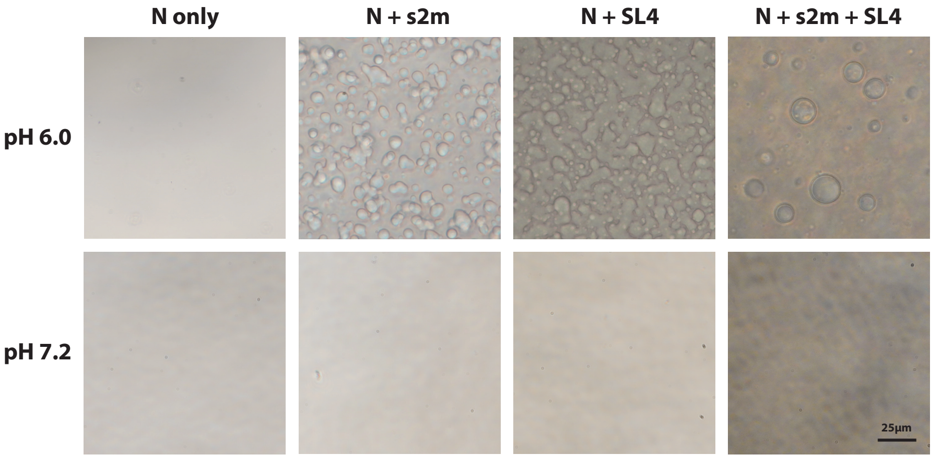

To adress the reviewer's concern about Figure 2D, we repeated the turbidity measurements in Pi buffer across the indicated pH range with N protein and 0.3 eq. SL4 RNA. Although the exact values are different for the two RNAs, both s2m and SL4 RNAs show the same pH-dependent LLPS behaviour with N protein in Pi buffer. We updated Figure 2D in the revised manuscript to include this additional data.

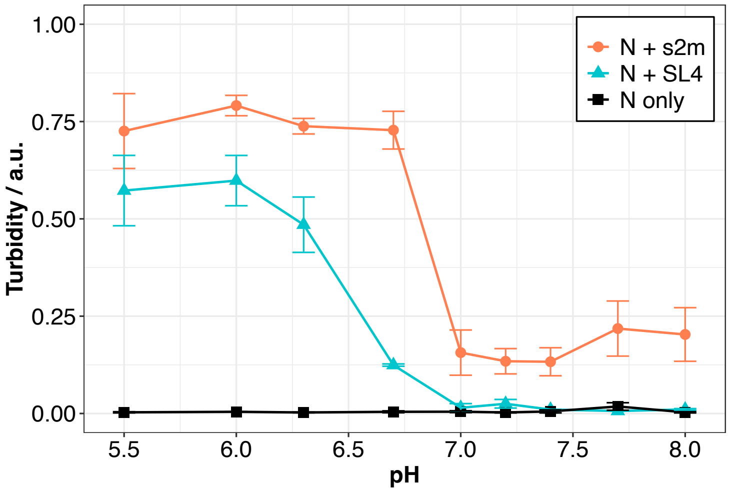

Lastly, regarding Figures 4B and 4C, for completion we repeated the experiments depicted in these two figures using SL4 RNA. In brief, the data with SL4 RNA matches what was observed for s2m RNA. The data using SL4 RNA is added to the revised manuscript as Supplemental Figure S8.

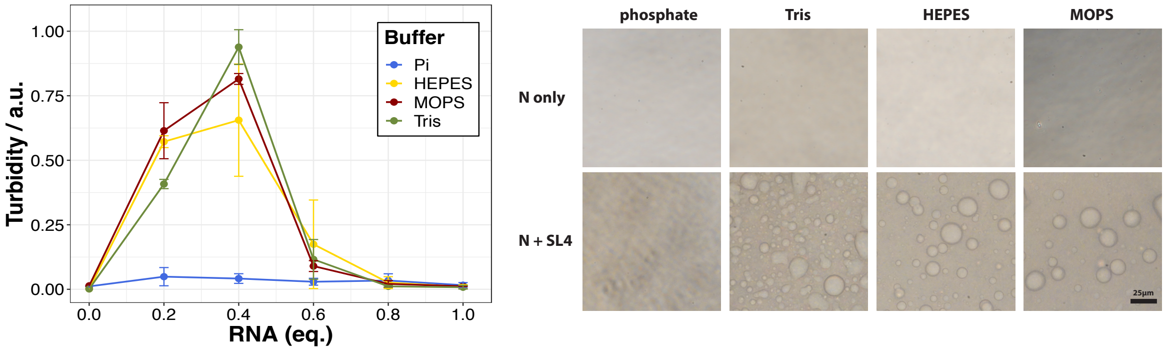

2) A helpful experiment related to point 1 above would be to test whether it is indeed charge that modulates phase separation. While the theoretical predictions in Figure 3A are helpful, the authors could use this as the basis for experimental probing of the effects of charge. The authors could probe the importance of each histidine by replacing them with lysine or arginine individually or in combination. By probing different combinations of positive charge on the surface of the domain, the authors can further probe N phase separation and deepen the understanding of specific surface charge contribution.

Thanks for this nice suggestion. We mutated all histidines individually to lysines to keep a positive charge at both pH 6.0 and 7.2, and repeated turbidity measurements with one fixed ratio of s2m or SL4 RNA in phosphate (Pi) buffer at pH 6.0 and pH 7.2. Our results show that the H356K mutant phase separates at both pH values, in contrast to wild-type (WT) protein or the other three mutants. In the WT protein, H356 is protonated at pH 6.0 but deprotonated at pH7.2. It seems that in Pi buffer, the addition of this single charge is essential to restore the protein's ability to undergo LLPS at pH7.2. We added this data to the revised manuscript as Figure 3D and extended the discussion.

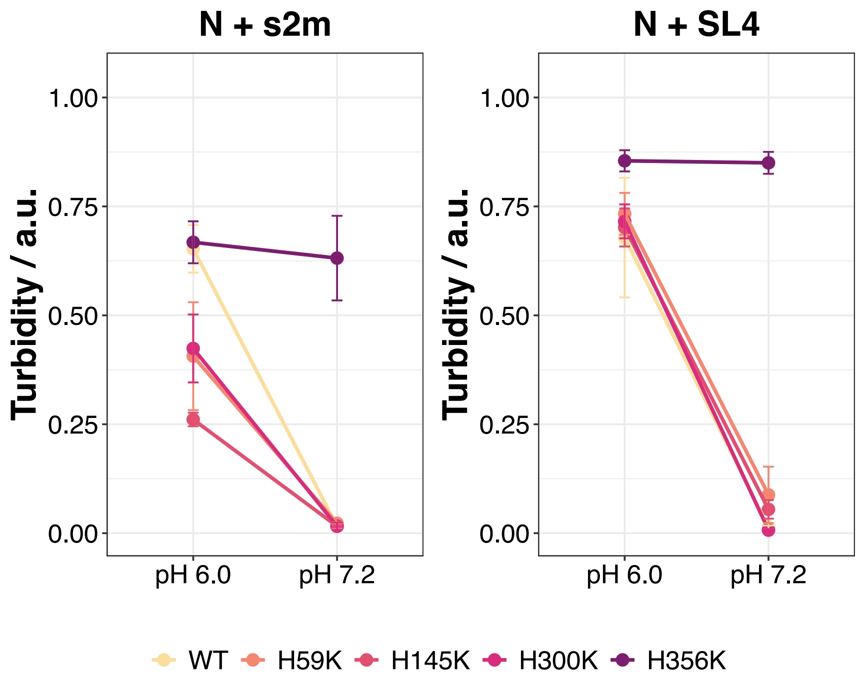

3) In the second line of the introduction, should "Mio" be "million?"
We thank the reviewer for spotting this. We meant indeed “million”. This is now corrected in the revised manuscript.

4) In the fourth and fifth line of the second paragraph of the introduction, please state that physiological pH is 7.2 to reduce potential confusion for the reader later in the manuscript.
We originally didn’t specify this as N protein has an isoelectric point (pI) of around 10 and is thus positively charged at all pH values below that, including pH values that classify as physiological. Furthermore, physiological pH is not limited to one specific pH value, but rather describes a range of pH roughly between 7.0 and 7.5. Since we are not describing our data in this paragraph, we choose not to specify an exact pH value here, but added a comment about the protein’s pI to the revised manuscript to clarify the matter.

5) In the fourth paragraph of the introduction, biophysical properties (e.g., the presence of IDRs in proteins), isn't as clear as it could be. IDRs are features of proteins while biophysical properties would include net charge, charge distribution, hydrophobic blocks, etc. Instead of presence of IDRs, the authors may want to state that net and local charge can be important biophysical characteristics of IDRs or domain surfaces. Doing so will lead more directly into their finding that the protonation of histidine residues is important for phase separation of N with RNA.
We thank the reviewer for spotting this. The statement is indeed a bit too vague as is. Thus, we updated the manuscript following the reviewer’s suggestions.

6) In the final paragraph of the introduction, please include the types of buffers used in the study so that the reader is aware of what the authors mean by "types of buffers." As written, this statement is too vague.
Thanks for the comment. We updated the manuscript with more specific information.

7) In Supplemental Figure S3, the position of the His residues on the surface of the domains is helpful. This figure could be made more impactful by including surface charge density with and without protonation of the His residues so that the reader can readily visualize how altering the charge of the His residues alters the local surface charge landscape.

We thank the reviewer for the nice suggestion. We rendered the surface charge distribution of both N2 and N4 domains at pH6.0 and pH7.2. The charge map nicely corroborates our NMR data, as H59 loses its positive charge upon shifting the pH from 6.0 to 7.2, while H145 and N300 remain neutral. Note that H356 is buried inside the structure of N4 domain and can thus not be seen. We added this figure as Supplemental Figure S5 to the revised manuscript.

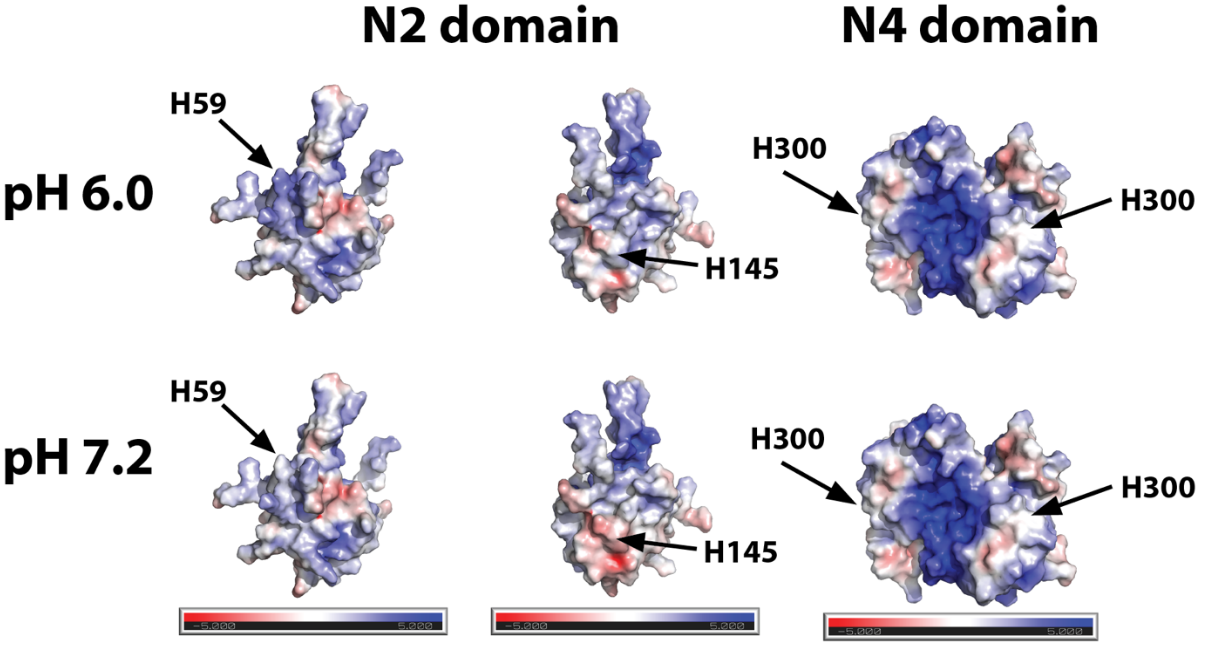

Based on the NTD surface charge data at pH 6 and 7.2 and the spectra we acquired with the wild-type and mutant N proteins, we could assign the four histidines.

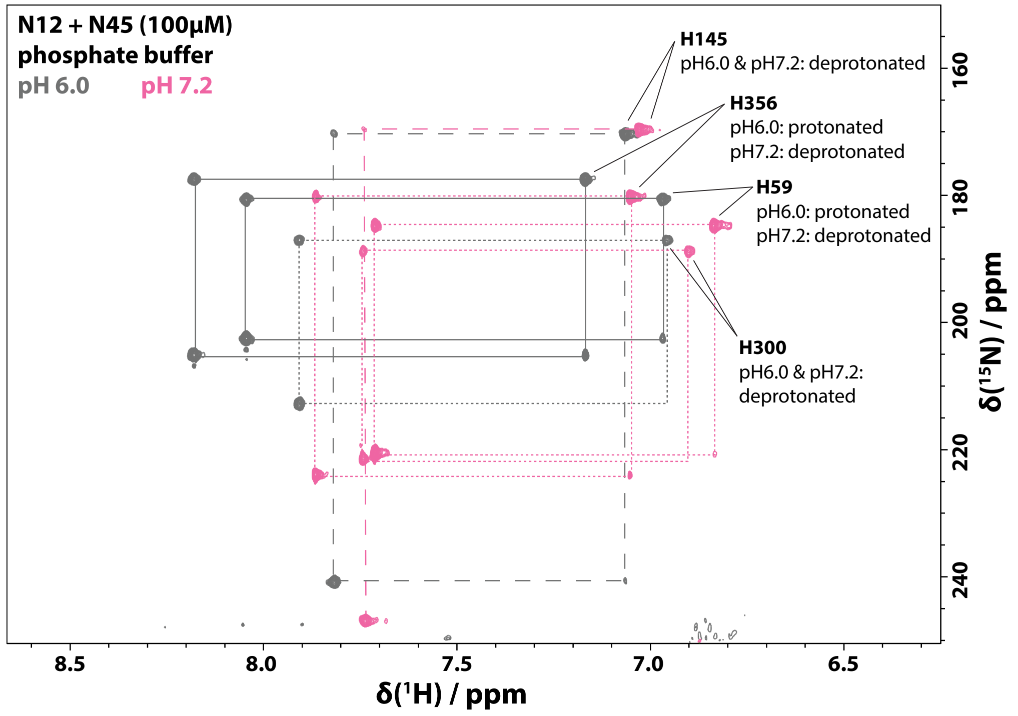

8) In the final paragraph of the results section "The type of buffer strongly influences LLPS of N protein with viral RNA" is stated. What is meant by "most intense" when referring to Tris-related phase separation? Is it that turbidity remains higher for Tris across a wider range of s2m concentrations? Or did the authors have something else in mind. When looking at the images in Figure 4C, it might be that phase separation occurs as readily in HEPES, perhaps even more. Clearly defining what is meant by "most intense" will be helpful.

Thanks for the comment; the expression "most intense" could indeed use some clarification. As the reviewer correctly assumed, we meant that turbidity remains high in Tris across a wider range of RNA concentration compared to HEPES or MOPS buffer. We now clarified this in the updated manuscript.

9) The final sentence of the results section states that "increased protein net charge..." alter phase behavior. This can be true, but another possibility should also be considered. Could it also be that increased local surface charge also can play a role? A charge map of the domains where the Histidine residues reside will be helpful for this point. If local charge may be important, perhaps the authors can also discuss this possibility.
This is indeed another possibility we need to consider, and nicely ties in with the suggestion the reviewer made in a previous point. We will discuss this possibility in the revised manuscript.

10) The discussion should be expanded to include a more detailed discussion of how these results relate to the complex cellular environment. In the third paragraph, the authors state that the intracellular pH can drop by as much as 0.4 units upon viral infection. This would mean that the pH drops from 7.2 to 6.8, which is still too high for phase separation of N + s2m based on Figure 1D. However, it could be that the crowding of the cell or general "buffer" characteristics of the nucleotides, proteins, salts, small molecules, etc. in the cytoplasm may promote N phase separation. It may also be that inclusion of SL4 would induce phase separation at this pH (see points 1 and 2 above for potential experiments to include). A more detailed discussion would provide the reader with a more complete picture of how this study contributes to the greater discussion of the relevance of in vitro assays to cellular behaviors.
We thank the reviewer for the very valuable suggestion. We expanded our discussion in the revised manuscript to address the above points.

Reviewer: 2

Understanding the molecular basis and physical rules of biomolecular condensation is currently the subject of extensive efforts in the community. Specifically, condensation is known to be affected by multiple variables, for instance salt concentration, pH, temperature, buffer composition, but untangling who does what between these different variables is not always obvious, precluding our understanding of this important biological phenomenon. In this study, Kathe and colleagues investigate this question in vitro using the Nucleocapsid (N) protein from SARC-CoV-2 as a model system and a variety of biochemical assays, including NMR.

Specifically, the authors shows that phase separation of N protein with RNA is strongly pH dependent, with a sharp decrease of condensation when pH gets higher than 6.7 in phosphate buffer. This does not depend on the ability of the protein to bind the RNA, implying that RNA binding does not necessarily correlates with the phase separated behaviour. Histidines are known to change charge around these pH values, and the authors indeed show experimentally that two histidines of the N protein get protonated when pH drops and thus change charge, therefore providing a rationale for the pH sensitivity of the phase separation behaviour. Then the authors make a very nice observation that there is an interplay between the pH and the buffer used to establish this pH with regard to phase separation. Indeed, they found that while a shift from pH 6 to 7.2 strongly inhibits phase separation in Phosphate buffer, it does not occur in Tris, MOPS or Hepes buffers, all robustly supporting phase separation at pH 7.2. As histidine protonation is the same at a given pH between the different buffers, this suggested an interaction effect between the buffer and the nucleocapsid protein. They then show experimentally that Tris and Phosphate buffers strongly interact with the N protein by NMR. They also measured the Zeta potential of the protein in the different buffers, which showed a

change of the effective charge of the protein (most positively charged in Tris buffer compared to neutral in phosphate buffer, all at a fixed pH of 7.2).

I find particularly interesting that given a constant pH and salt concentration, the choice of the specific molecule used to buffer the solution profoundly impacts the ability of the SARS-CoV-2 nucleocapsid to phase separate, This of course makes perfect sense with known biochemistry given the effective charge of the buffer molecule, which can (or not) shield the charged residues at the surface of the nucleocapsid. But I think it's a very nice example where the buffer choice matters and should help the community interpreting/designing their experiments. Figures are nicely rendered and methods are adequate in my opinion. I recommend publication, provided the author address my (minor) comment bellow.

We thank the reviewer for the positive opinion and constructive comments.

My main comment about this paper is that I don't think the buffer the authors used can "to mimic the physiological conditions within a cell", page 3. Their buffer only has 50 mM NaCl and so has an osmolarity that is twice less than that of a cell. I think this matters, as salt concentration is also known to affect phase separation (plus the whole paper is about charges). Obviously, I'm not suggesting the authors redo all their experiments with a higher salt concentration (the set of experiments presented is all coherent and I think support the claims of the paper). But if the authors want to keep that specific sentence, they should at least do one control showing that a physiological salt concentration does not affect the ability of the N protein to phase separate in the presence s2m upon pH change. I think keeping this relation to physiological conditions is important, especially because the authors make a very nice point in discussion that intracellular pH drops upon infection, which could favour phase separation, which I think is a cool idea (it might be related to some reports that lowering cell culture pH improves the production of lentiviral and retroviral vectors, Holic et al. Human gene therapy clinical development, 2014).
This is indeed a very valid point. When choosing the salt concentration for our buffers, we used intracellular sodium concentrations as a reference, although that doesn't reflect the total salt content or osmolarity in a cell, as pointed out by the reviewer.

Thus, we performed the suggested control experiment by measuring the turbidity of N protein with 0.3 molar equivalents SL4 RNA in phosphate (Pi) buffer at both pH6.0 and pH7.2. We compared our original buffers with 50mM NaCl (dark blue) to new buffers containing 100mM NaCl (light blue). As can be appreciated from the figure below, the LLPS behaviour is identical at both NaCl concentrations and also follows the same pH dependency as observed previously. This figure will be added to the manuscript as Supplemental Figure S2.

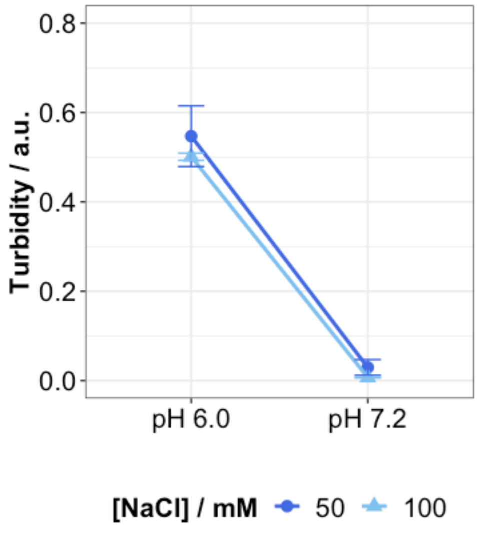

We would like to conclude by thanking again all the referees for their valuable and constructive comments. Furthermore, we are also grateful for the very positive Editor comments. We believe that our clarifications and additions improved the quality and clarity of the manuscript, and that our revised document is now suitable for publishing in Molecular Biology of the Cell.

Sincerely and on behalf of all authors,

Frédéric Allain

RE: Manuscript #E23-12-0500R
TITLE: "Buffer choice and pH strongly influence phase separation of SARS-CoV-2 Nucleocapsid with RNA"

Dear Dr. Allain:

I am pleased to accept your manuscript for publication in Molecular Biology of the Cell.

The revisions performed by the authors have adequately addressed the concerns of the reviewers.

Sincerely,
Jeffrey Woodruff
Monitoring Editor
Molecular Biology of the Cell

Dear Dr. Allain:

Congratulations on the acceptance of your manuscript! Thank you for choosing to publish your work in Molecular Biology of the Cell (MBoC).

Within 10 days, an unedited PDF of your manuscript will be published on MBoC in Press, an early release version of the journal. The date your manuscript appears on this site is the official publication date.

Your copyedited and typeset manuscript will be scheduled for publication in the next available issue of MBoC. Our production team will notify you when the page proofs of your paper are ready for your review.

MBoC offers the option to publish your paper with immediate open access. Open access can increase the discoverability and usability of your research. If you would like to publish your paper with immediate open access but did not select this option during initial submission, please contact the MBoC Editorial Office (mbc@ascb.org).

We invite you to submit images related to your accepted manuscript to be considered for the journal cover.
Cover images must contain color, TIF Format
Size image at 7.4" wide x 5.85" deep
RGB and CMYK color (please submit two separate files, one in each format)
300 dpi resolution

Please also send a 1-paragraph description of the image that a general audience could understand. Include with the caption the name and institution of the individual(s) the image should be attributed to. Please provide this description in a Word file. You can send the file via email or by your favorite file transfer service to mboc@ascb.org.

Authors of Articles and Brief Communications are also encouraged to create a short video abstract to accompany their article when it is published. These video abstracts, known as Science Sketches, are up to 2 minutes long and will be published on YouTube and then embedded in the article abstract. Science Sketch Editors on the MBoC Editorial Board will guide you as you prepare your video. Information about how to prepare and submit a video abstract is available at www.molbiolcell.org/science-sketches. Please contact mboc@ascb.org if you are interested in creating a Science Sketch.

We look forward to publishing your paper in MBoC.
Sign up for MBoC's new content alerts here https://tinyurl.com/3bcsyk3e

Sincerely,

Eric Baker
Journal Production Manager
MBoC Editorial Office
mbc@ascb.org
